# Unlocking the Potential of Food Waste: A Review of Multifunctional Pectins

**DOI:** 10.3390/polym16182670

**Published:** 2024-09-22

**Authors:** Marta Tsirigotis-Maniecka, Ewa Górska, Aleksandra Mazurek-Hołys, Izabela Pawlaczyk-Graja

**Affiliations:** Laboratory of Bioproduct Technology, Faculty of Chemistry, Wroclaw University of Science and Technology, Wyb. Wyspianskiego 29, 50-370 Wroclaw, Poland; ewa.gorska@pwr.edu.pl (E.G.); aleksandra.mazurek@pwr.edu.pl (A.M.-H.); izabela.pawlaczyk@pwr.edu.pl (I.P.-G.)

**Keywords:** food waste, emerging separation technologies, pectins, polyelectrolyte, biomaterial, hydrogel, interfacial properties, rheology, techno-functional properties, biological activity

## Abstract

This review comprehensively explores the multifunctional applications of pectins derived from food waste and by-products, emphasizing their role as versatile biomaterials in the medical-related sectors. Pectins, known for their polyelectrolytic nature and ability to form hydrogels, influence the chemical composition, sensory properties, and overall acceptability of food and pharmaceutical products. The study presents an in-depth analysis of molecular parameters and structural features of pectins, such as the degree of esterification (DE), monosaccharide composition, galacturonic acid (GalA) content, and relative amounts of homogalacturonan (HG) and rhamnogalacturonan I (RG-I), which are critical for their technofunctional properties and biological activity. Emphasis is placed on pectins obtained from various waste sources, including fruits, vegetables, herbs, and nuts. The review also highlights the importance of structure–function relationships, especially with respect to the interfacial properties and rheological behavior of pectin solutions and gels. Biological applications, including antioxidant, immunomodulatory, anticancer, and antimicrobial activities, are also discussed, positioning pectins as promising biomaterials for various functional and therapeutic applications. Recalled pectins can also support the growth of probiotic bacteria, thus increasing the health benefits of the final product. This detailed review highlights the potential of using pectins from food waste to develop advanced and sustainable biopolymer-based products.

## 1. Introduction

**Figure 1:**
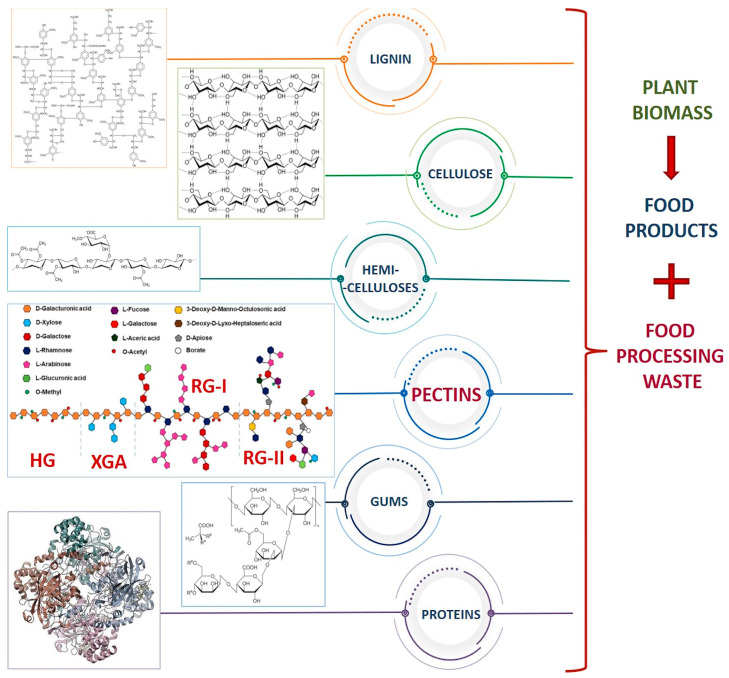
Biopolymers present in food processing waste.

The comprehensive and overall approach to waste valorization is expected to increase the importance of waste management in the food industry with an equal contribution to wealth generation through the production of multifunctional materials [1,2,3]. According to the Food and Agriculture Organization (FAO) (2022), approximately one-third of the world’s food is wasted every year. Among the wasted products, plant materials, such as root tubers, oil plants, and by-products of fruit and vegetable processing, have a greater share. The FAO of the United Nations estimated that global agricultural production produces about 5 billion tons of crop residues per year [4]. However, the actual amount of agro-waste is difficult to quantify due to the absence of comprehensive data in many countries. Such waste contains significant amounts of valuable ingredients that can be used to benefit the food processing industry and reduce environmental problems such as global warming. It is well known that the edible parts of fruits, vegetables, nuts, and herbs are filled with metabolites of numerous health-promoting properties, such as polyphenols, carbohydrates, proteins, organic acids, vitamins, pigments, minerals, etc. (Figure 1). On the other hand, the nonedible parts of these plants, such as peels, post-processing pulp, roots, husks, leaves, and other residual fractions, seem to be a poorly used source of compounds that are equally valuable as those mentioned above [1,2]. The key motivation behind the valuation of this type of biomass is continuous access while maintaining raw materials with sufficient productivity, which results from the production of municipal waste. The versatility in structure and function of biomass waste from the food industry predetermines it to be considered a feedstock for the production of numerous biopolymers, mainly: cellulose, hemicellulose, and lignin [5]. So far, corn, potato peels, tomato pomace, carrot peel and its pomace, sunflower heads, grape pomace, apple pomace, pomegranate peels, watermelon rinds, and mango peels have been a great source of cellulose and its derivatives [1]. However, the interest has recently moved from cellulose to the next-generation biopolymers identified in waste products. For example, many agricultural waste by-products contain water-soluble polysaccharides with unique structures and properties, i.e., pectins.

Currently, the perception of pectins extends far beyond dietary fiber. Pectins are considered as a multifunctional and safe material platform for use as an additive in functional foods, nutrition restoration, drug-delivery systems, disease treatment, and tissue engineering [6,7]. Since these polysaccharides are a versatile biomaterial, they exert a significant effect on the chemical composition and physical properties of food and pharmaceutical products, enhance sensory properties and general customer acceptability, and they can even facilitate the growth of probiotic bacteria in the final formulation [3]. Moreover, these biopolymers are eco-friendly, biodegradable, and biocompatible. These characteristics contribute to pectins’ exceptionally high nutritional and economic value [2,3].

Pectins are present in primary cells and middle lamellas and, in combination with cellulose and lignin, constitute the plant cell wall [8]. From a chemical point of view, pectins are heteropolysaccharides composed of α-(1-4)-D-galacturonic acid (GalA) and neutral monosaccharides. Within the pectic chain, there are three sub-domains: (i) homogalacturonan (HG), which is the poly-α-(1–4)-D-galacturonic acid units’ backbone, referred to as a linear or “smooth” region; (ii) rhamnogalacturonan of type I (RG-I), which is composed of repeating units of α-(1–2)-L-rhamnosyl-α-(1–4)-D-galacturonosyl substituted with side chains of mainly α-L-arabinofuranose and α-D-galactopyranose units, which are referred to as the branched or “hair”’ region; (iii) rhamnogalacturonan of type II (RG-II), where the backbone contains seven-to-nine α-(1-4)-D-galacturonic acid units and up to six side chains formed mainly with L-galactose, L-fucose, and rare monosaccharides (D-apiose, D-glucuronic acid, and L-aceric acid), which is also called the branched or “hairy” region [9,10]. HG and RG-I domains are the most abundant in plant cell walls, but the structure and proportions of HG, RG-I, and RG-II vary from source to source. A higher HG/RG-I ratio is considered to be an indicator of the linearity of pectin [11,12]. GalA moieties are often C-6 methyl esterified (usually in HG domains) and/or C-2/C-3 O-acetylated, and, following the degree of esterification (DE), these biopolymers are classified as high-methoxyl pectin (DE > 50%) (HMP) and low-methoxyl pectin (DE < 50%) (LMP) [8]. The level of esterification is one of the most important structural features as it affects the properties and behavior of pectins under various conditions and often directly determines their functionality, i.e., along with increasing DE chain stiffness decreases [13], as low pH-value HMPs are characterized with less electrophoretic mobility and occur in a more coiled form (lower hydrodynamic radius) than LMPs [13,14].

A detailed analysis of the correlation between functional and structural properties is one of the key factors in the production of multifunctional pectins, which is necessary to fully exploit the potential of the commonly available biomass. This article presents a review of numerous pectins only with scientifically proven functionality and obtained from food waste and by-products over the last 10 years. All mentioned pectins were thoroughly characterized in terms of the extraction method (in brief), structural features (DE and monosaccharide composition, including GalA content, HG/RG-I ratio, and Mw), and their functionality in terms of the mechanisms behind the physicochemical interaction and/or biological activity. This unique approach provides a wide understanding of the possibilities and advantages of using pectins as advanced biomaterials and promotes a circular economy. To improve readability, the data have been organized by waste type: fruits (Table 1), vegetables (Table 2), and herbs and nuts (Table 3).

## 2. Comparative Analysis of Pectin Structure with Recovery Technology

Pectins can be commercially extracted from many agricultural by-products, including citrus peels, banana peels, mango peels, apple pomace, sugar beet, cocoa husks, mulberry branch bark, broad bean peels, sisal waste, watermelon waste, pomegranate peels, and passion fruit peel waste. Regardless of the type and chemical structure, pectin is obtained by the extraction process. Indeed, the extraction of pectin from various types of food and agricultural by-products and wastes has been practiced for many years. In these processes, the main objective is to extract pectin with the highest possible yield and highest purity. However, conventional extraction (CE) has several limitations, such as thermal degradation, undesirable physicochemical and functional properties, and a low degree of esterification due to prolonged direct heating in comparison to emerging processing technologies (EPT). In addition, the plant cell wall is composed of various polysaccharides and structural proteins, which makes the CE of pectins a challenging task. The amount of pectin that can be extracted depends on the extraction method and various related parameters. Therefore, researchers have been investigating the possibility of solving CE problems through EPT, including emerging thermal and non-thermal technologies [15].

**Figure 2:**
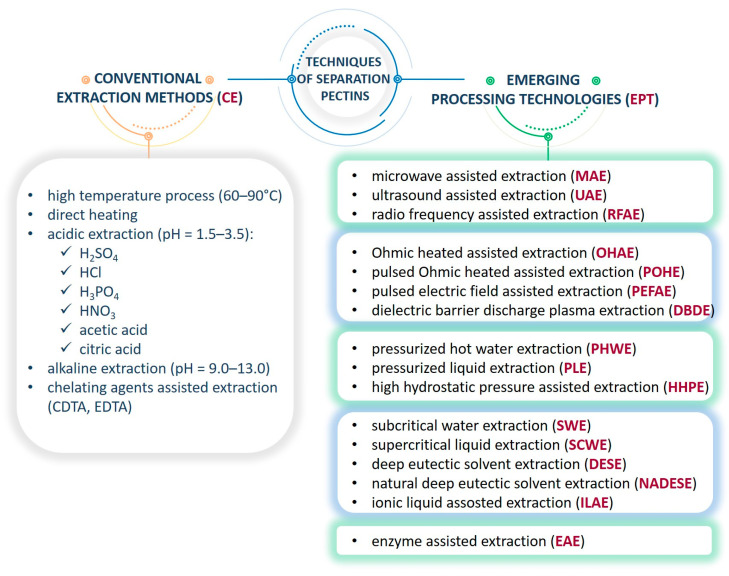
The methods of pectin extraction.

Currently, the vast majority of research is focused on refining the technology of isolating pectins from plant material to maximize the process efficiency and quality of the final product, and less emphasis is put on obtaining pectins with a specific chemical structure [16,17]. Among the multitude of extraction technologies, the one oriented on a specific type of biopolymer might be selected (Figure 2), e.g., those employing environmentally friendly solvents (natural deep eutectic solvents (NADES) or ionic liquids or supercritical fluids (scCO_2_ or scH_2_O)) [16,18]. The extensively promising novel techniques are: microwave-assisted extraction (MAE), ultrasound-assisted extraction (UAE), pressurized hot water extraction (PHWE), pressurized liquid extraction (PLE), pulsed electric field-assisted extraction (PEFAE), ohmic heated-assisted extraction (OHAE), pulsed ohmic heated-assisted extraction (POHE), enzyme-assisted extraction (EAE), dielectric barrier-discharge plasma extraction (DBDE), and high hydrostatic pressure-assisted extraction (HHPE). Most of these techniques have been established, and a few, like pulsed ohmic extraction and high-pressure extraction, are on the verge of commercialization [19,20]. Each of these techniques is characterized by different advantages and disadvantages, i.e., hot acid extraction is fast, economically favorable, and a well-defined process, but under acidic conditions, the structural integrity of the pectins might be affected and carries a corrosive hazard. Microwave- or ultrasound-assisted extraction is safer than conventional extraction techniques, but they are less cost-effective. Generally, EPT is definitely a promising alternative to CE because it reduces the use of toxic organic solvents and generates minimal waste, shortens processing time, enables selective extraction, and reduces energy consumption and associated costs, all while providing satisfactory results [21]. Moreover, tailoring the process conditions, such as pH, ionic strength, temperature, time, physical factors, etc., the final pectin often meets specific requirements in terms of structure, namely: GalA content, DE, the HG/RG-I ratio, and molecular weight (Mw) [17,18]. For the efficient recovery of such polyelectrolytes, many alternative processing methods, such as low-cost and efficient industrial processing, have been widely considered.

**Figure 3:**
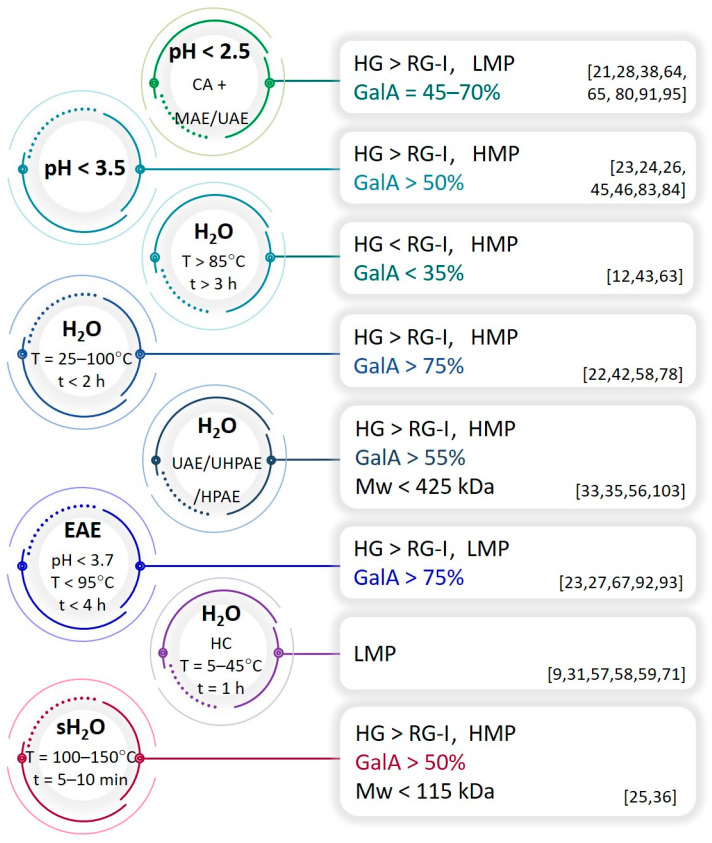
The influence of the condition of pectin-extraction process on its chemical structure (CA—citric acid solution as extraction medium; MAE—microwave-assisted extraction; UAE—ultrasound assisted-extraction; EAE—enzyme-assisted extraction; HPAE/UHPAE—high-pressure/ultra-high pressure-assisted extraction; HC—hydrodynamic cavitation; sH_2_O—subcritical water).

To effectively extract pectins with specific properties from plant biomass, the process parameters must be carefully selected. The first step in the process is to disrupt the plant cell wall to allow the solvent to penetrate the cell, dissolve the substances of interest, and transfer them outside the cell by mass transport phenomenon. Separation of acidic heteropolysaccharides can be performed by chemical, physical, or enzymatic treatment with organic or inorganic solvents [22]. Mild conditions, such as hot/room-temperature water extraction, allow one to extract loosely bound branched pectins from the cell wall, while more aggressive conditions, such as the hot acid technique, are required to extract linear pectins that are tightly attached to the cell wall [12,23]. Moreover, prolonged exposure of pectins to heat, acid, or physical factors can lead to their degradation and reduction of molecular weight. Therefore, the time of the isolation process should also be carefully considered. The structural diversity of pectins is known to depend not only on the plant material but also on the extraction technique. Following different procedures, pectins with different structural characteristics in terms of Mw, GalA content, DE (%), and HG/RG-I proportion can be extracted from a single plant material. It should be noted that slight discrepancies in terms of these features may result from the biomass batch and not necessarily from the extraction conditions. Figure 3 summarizes the approximate structural characteristics of pectins extracted from various waste biomass, i.e., fruits, vegetables, nuts, herbs, etc. In fact, it is very difficult to draw more precise characteristics of these substances according to separation methods because the cell walls of individual plant tissues (fruit pulp, peels, leaves, stems, pods, husks, etc.) differ in their function, which is reflected in the structure of polysaccharides.

The most important criterion for selecting these factors is to maximize recovery efficiency and minimize the risk of pectin degradation. For non-laboratory-scale processes, high energy efficiency and negligible negative environmental impact of the process should also be considered. Therefore, the main objective proposed in this comparison is to investigate the possibility of isolating pectins with previously studied and confirmed functional properties and/or biological activity.

### 2.1. Overview of the Structural Features of Pectins Extracted from Fruit Biomass

In general, the most abundant pectin industrial waste is fruits, especially peels and pomace. Fruit-derived pectins differ greatly in chemical structure, which, in fact, determines their functional properties and biological activity, which are carefully described in the following sections. The range of techniques used to extract pectins from these raw materials is enormous and diverse to a great extent, including conventional extraction methods (CE) and emerging processing technologies (EPT). An ideal example that illustrates the influence of the extraction medium and method used on the chemical structure of polysaccharides isolated from fruits is those obtained from jackfruit peel and seeds, watermelon rinds, lemon peel, orange peel, apple pomace, and wild strawberry leaves. Jackfruit peels and seeds treated with hot sulfuric acid (pH = 1.5) [24] or under the influence of ammonium oxalate (T = 85 °C, pH = 4.6) [24] provided HMPs of lower molecular weight (Mw < 100 kDa) (LMw), while peels treated with hot citric acid (pH = 2.0) or scH_2_O provided HMPs of higher molecular weight (Mw > 100 kDa) (HMw) [25]. Watermelon peels, when treated with hot inorganic acid alone or followed by enzymatic treatment, resulted in LMw-HMP [26,27], but when the rinds were treated with ultrasound-assisted hot organic acid, they provided HMw-HMP [28]. Lemon peels provided HMw-HMP by hot citric acid extraction, followed by enzymatic modification [29]. Furthermore, this waste by-product provided LMw-LMP via hot water extraction [30], HMw ultralow methoxylated pectins via the hydrodynamic cavitation method (HC) [31], and microwave-assisted citric acid extraction [21]. LMw-HMP of the orange peel was extracted with hot citric acid [32], while HMw-HMPs were obtained with high-pressure-assisted extraction (HPAE) [33] or ultrahigh-pressure-assisted extraction (UHPAE) [34]. Apple pomace, when treated with scH_2_O, provided LMw-HMPs [35], but when treated with hydrochloric acid and ultrasounds or microwaves, it provided HMw-HMPs [36]. The same waste by-product under the influence of a strong reducing agent (NaBH_4_) provided extremely low methoxylated pectins [37], while under microwave-assisted citric acid, it provided LMw-LMP [38]. Wild strawberry leaves under the influence of alkali provided only HMw-LMPs regardless of the CE or EPT applied. The interesting thing is that wild strawberry leaf HMw-LMPs were characterized with different uronic acid (UA) content, which were reflected significantly in the polymer functionality. Considering the UA in the structure, these pectins can be arranged in the following series: room-temperature alkaline-extracted LMw-HMPs < ultrasound-assisted alkaline-extracted LMw-HMPs < hot alkaline-extracted LMw-HMPs < ultrafiltration-assisted alkaline-extracted LMw-HMPs < microwave-assisted alkaline-extracted LMw-HMPs [39,40].

Although CEs are more environmentally demanding, they are still the starting point for obtaining pectins with useful properties. Extraction with water at a temperature near the boiling point (T > 90 °C) resulted in HMPs of red chilto fruit [41], palmyra palm [42] and gabiroba [43] pulp, and blackberry leaves [44]. The HMPs were also extracted with hot inorganic acid (T > 85 °C, pH ≤ 2.0) from banana passion fruit [45] and bigarade fruit [46] peels, as well as with hot organic acid (T > 70 °C, pH = 1.8–3.0) from the fig fruits stalks [47] and the dragon fruit peel [48]. Meanwhile, LMPs were derived from kinnow fruit [49], mangosteen fruit rinds [50], melon fruit peel [51] by hot water extraction, and from chocolate wine fruit peel [52] with hot inorganic or organic acid extraction (T > 85 °C, pH < 4.4). Hot acid chelating agent-assisted extraction (T = 70 °C, EDTA, pH = 3.0) resulted in LMP from the prickly pear cactus fruit peel [53].

EPT provided polysaccharides of desirable structural properties from various fruit waste by-products without the necessity of excessive energy consumption as CE methods. EPT procedures take 1–60 min [42,54] instead of a couple of hours of treatment, which is typical for CE techniques. Recently, the potential of extraction conducted in aqueous solutions under ambient conditions seems to be efficient, especially when the disruption of plant cells and polysaccharide mass transfer is enhanced with a physical factor, such as ultrasounds and/or microwaves. Room-temperature water extraction provided papaya fruit pulp HMP [55] and kiwifruit pomace pectin [22], while water-UAE provided grapefruit peel HMP [56]. LMPs were derived from grapefruit albedo by HC-assisted water extraction [57,58,59] and formed a melon peel by water-MAE [60]. Acid-UAE allowed one to obtain the LMP of a banana peel (sulfuric acid, pH = 1.5) [61] and the HMP of a mango peel (citric acid, pH = 2.5) [62]. Acid-MAE provided LMPs of a banana peel (hydrochloric acid, pH = 3.0) [63] and pineapple peel (citric acid, pH = 2.0) [64]. A combination of ultrasounds and microwaves when citric acid (pH = 1.4) was used as an extraction medium allowed the receipt of LMP of the fig peel [65], while in the case of HMP of the passionfruit peel, sulfate ammonium (pH~5.0) was used as an extraction medium [66]. EPT also includes extraction techniques using “green solvents”, which have been proven to be effective in obtaining pectins from fruit waste. NADES (choline chloride:maltose) was used to extract LMP from the kinnow peel [8], and scH_2_O was used to extract HMP from the mandarin peel [35]. Finally, the enzyme treatment under acidic conditions of plant cells resulted in the LMP of the yuzu peel [67] and pectin of kiwifruit pomace [22].

To summarize the influence of extraction conditions on the properties of pectins isolated from fruit waste via CE, the following dependencies were observed: (i) hot water extraction favors pectins of HG >> RG-I, moderate-to-high DE (37–73%), moderate-to-high GalA content (45–84%), and Mw > 64 kDa [30,41,42,49]; (ii) hot mineral acid extraction favors pectins of HG > RG-I, moderate-to-high DE (48–80%), high GalA content (62–86%), and Mw > 34 kDa [24,26,45,46,68]; (iii) hot organic acid extraction favors pectins of HG > RG-I, high DE (60–74%), high GalA content (57–82%), and Mw > 174 kDa, which is more branched than described in (ii) [22,25,32,47]; (iv) hot alkaline extraction favors pectins of HG > RG-I, low DE (~10%), high GalA content (57–68%), and Mw 6–350 kDa [39,40]. In the case of EPT, processes employ many physical factors that influence the properties of separated pectins. Thus, the summary is more complex: (i) cold water extraction favors pectins of HG >> RG-I, high DE (~60%), high GalA content (74–83%), and Mw > 2 kDa [22,55]; (ii) high-pressure water extraction favors pectins of HG >> RG-I, high DE (57–71%), moderate-to-high GalA content (53–69%), and Mw > 306 kDa [33,34,69]; (iii) UAE favors pectins of HG > RG-I, moderate-to-high DE (44–89%), high GalA content (57–68%), and Mw > 270 kDa [28,36,56,62,66]; (iv) HC-assisted extraction favors LMPs [70,71]; (v) MAE favors pectins of HG > RG-I, low-to-moderate DE (5–65%), moderate-to-high GalA content (33–80%), and Mw > 0.5 kDa [21,36,38,39,60,64,65]; (vi) scH_2_O-assisted extraction favors HG > RG-I, high DE (61–86%), high GalA content (52–91%), and Mw 53–113 kDa [25,35]; (vii) EAE favors pectins of HG >> RG-I, high GalA content (81–85%), and Mw 50–670 kDa [22,27,29,67].

**Table 1:** Overview of extraction conditions, main structural features, and functionality of pectins from selected waste and by-products of fruits.

Plant (Fruit)	Waste By-Product	Extraction Technique	Conditions of Extraction					$\frac{HG}{RGI}$	DE (%)	GalA (%)	Mw (kDa)	Utility	Ref.
			Medium	pH	T (°C)	t	Factor						
*Actinidia chinensis* (kiwifruit)	Pomace	CE	C_6_H_8_O_7_	2.2	50	1 h	-	6.2	n. a.	82.4	840	Shear-thinning property	[22]
		CE	H_2_O	3.7	25	30 min	-	5.3	n.a.	82.7	850	Shear-thinning property	
		EAE	H_2_O	3.7	25	30 min	CEL PG EAR	5.8	n.a.	84.6	670	Shear-thinning property	
*Akebia* *Trifoliata* (chocolate wine)	Fruit peel	CE	C_6_H_8_O_7_	2.2	85	2 h	-	n.a.	29.4	79.9	112	Biomaterial for sponges; emulsifying activity; gel-forming property; reductant of AgNO_3_ to Ag nanoparticles	[52]
*Ananas comosus* (pineapple)	Fruit peel	MAE	C_6_H_8_O_7_	2.0	85	10 min	1400 W	n.a.	39.4	44.8	889	Antioxidant activity; Film-forming property	[64]
						30 min	420 W						
*Artocarpus heterophyllus* (jackfruit)	Fruit peel	CE	C_6_H_8_O_7_	2.0	90	2 h	-	0.9	73.9	57.0	174	Gel-forming property; Shear-thinning property	[25]
		SWE	H_2_O	-	138	9.15 min	-	1.0	61.1	52.3	113	Gel-forming property; Shear-thinning property	
	Fruit peel and seeds	CE	(NH_4_)_2_C_2_O_4_	4.6	85	1 h		0.7	78.3	63.3	40	Gel-forming property	[24]
	Fruit peel and seeds	CE	H_2_SO_4_	1.5	90	1.5 h	-	1.1	63.0	62.7	39	Gel-forming property	
*Borassus aethiopum* Mart. (palmyra palm)	Fruit pulp	CE	H_2_O	~5.3	90	30 min	-	24.8	73.2	84.0	338	Emulsifying activity; Gel-forming property	[42]
*Campomanesia* *xanthocarpa* (gabiroba)	Fruit pulp	CE	H_2_O	-	120	4 h	-	0.5	60.0	33.5	~1000	Antitumor activity	[43]
*Carica papaya* L. (papaya)	Fruit pulp	CE	H_2_O	-	25	20 min	-	3.2	59.0	74.8	102 2	Antitumor activity	[55]
*Citrullus lanatus* (watermelon)	Fruit rinds	CE	HNO_3_	1.0	100	1 h	-	2.8	63.0	74.2	34	Emulsifying activity; foaming activity; shear-thinning property	[26]
		UAE	C_6_H_8_O_7_	1.8	-	43 min	573 W	1.9	44.1	69.0	271	Emulsifying activity	[28]
		CE followed by enzymatic treatment	H_2_O	1.4	95	1.5 h	-	3.8	4.8	81.8	50	Biomaterial for hydrogel beads and aerogel beads	[27,72]
			PBS	4.0	40	24 h	EAR PME						
*Citrus* *reticulata* (mandarin)	Fruit peel	SWE	H_2_O	-	100	5 min	-	11.3	71.9	91.0	63	Antioxidant activity; anti-tumor activity; gel-forming property; shear-thinning property	[35]
*Citrus* *junos* (yuzu)	Fruit peel	CE followed by enzymatic treatment	C_6_H_8_O_7_	1.6	80	4 h	-	5.9	34.6	83.5	n.a.	Biomaterial for hydrogel beads	[67]
			H_2_O	4.5	45	3 h	PME						
*Citrus aurantium* (bigarade)	Fruit peel	CE	HCl	1.5	90	2 h	-	6.8	80.3	86.0	80	Emulsifying activity; Shear-thinning property	[46]
*Citrus limon* (lemon)		HCAE	H_2_O	-	6–45	1 h	-	n.a.	8.0	n.a.	n.a.	Antibacterial activity; neuroprotective activity	[31,70,71]
		MAE	C_6_H_8_O_7_	1.5	-	3 min	700 W	1.3	5.8	60.0	616	Antioxidant activity; emulsifying activity	[21]
		CE	H_2_O	1.8	85	30 min	-	5.1	48.2	52.5	82 64	Prebiotic properties	[30]
		EAE	C_6_H_8_O_7_	3.5	50	4 h	CEL PL	5.2	79.1	83.1	225	Gel-forming property; stabilizing ability of food and pharmaceutical products	[29]
*Citrus nobilis* x *Citrus deliciosa* hybrid (kinnow)	Fruit peel	CE	H_2_O	5.0	90	30 min	-	n.a.	37.2	47.7	652	Shear-thinning property;	[49]
		NADESE	choline chloride: maltose 5:2	-	75	4 h	-	n.a.	36.8	78.2	n. a.	Emulsifying activity;	[8]
*Citrus paradisi* (grapefruit)	Fruit albedo	HCAE	H_2_O	-	7.5–38	1 h	150 W	n.a.	14.0	n. a.	n. a.	Anti-apoptotic activity; Antimicrobial activity; antioxidant activity; antitumor activity; cardioprotective effect; immunomodulatory activity;	[9,57,58,59]
		UAE	H_2_O	-	37	28 min	61.5 W	1.2	58.8	56.4	279	Antioxidant activity; lipase-inhibitory property; shear-thinning property	[56]
*Citrus sinensis* Osbeck (orange)	Fruit peel	CE	C_6_H_8_O_7_	3.0	100	10 min	-	n.a.	>60.0	81.2	2	Antimicrobial activity; antioxidant activity; composite film-forming ability	[32]
		HPAE	H_2_O	-	-	15–30 min	125–500 MPa	6.2–9.3	57.1–63.3	87.7–92.6	374–422	Emulsifying activity; gel-forming property	[33]
		UHPAE	H_2_O	-	55	10 min	550 MPa	n.a.	71.0	63.0	306	Anti-diabetic property; cholesterol-regulating property	[34,69]
*Citrus unshiu* Marc (Satsuma mandarin)	Segment material	CE	processing waste water (HCl_aq_) discharged from citrus canning process					1.1	48.8	45.0	531	Obesity-mitigating agent; prebiotic activity	[68,73,74]
*Cucumis melo* L. (melon)	Fruit peel	MAE	H_2_O	-	-	13 min	414 W	1.0	19.3	40.7	57	Antioxidant activity; emulsifying activity; foaming capacity	[60]
		CE	C_6_H_8_O_7_	1.0	95	3.3 h	-	n.a.	15.0	48.0	68	Emulsifying activity;	[51]
*Ficus carica* L. (fig)	Peel	UAE followed by MAE	C_6_H_8_O_7_	1.4	70	21.3 min	70 W	1.8	33.6	55.4	6890	Antioxidant activity; antitumor activity	[65]
						11.7 min	580 W						
	Fruit stalks	CE	C_6_H_8_O_7_	1.8	95	1 h	-	3.6	65.9	63.0	n.a.	Film-forming ability	[47]
*Fragaria vesca* L. wild strawberry	Leaves	CE	NaOH	13.0	room	24 h	-	0.6	18.4	24.5	14–350	Anticoagulant activity	[39]
		CE	NaOH	13.0	100	6 h	-	1.0	11.1	57.6	25–350	Anticoagulant activity	
		UAE	NaOH	13.0	25	40 min	60 W	1.4	18.6	40.6	2–160	Anticoagulant activity	
		MAE	NaOH	13.0	80	20 min	200 W	5.5	12.5	79.5	12–160	Anticoagulant activity	
		UF	NaOH	13.0	100	6 h	PES 1 Bar	2.2	n.a.	68.1	6–180	Anticoagulant activity	[40]
*Garcinia mangostana* (mangosteen)	Fruit rind	CE	H_2_SO_4_	2.0	90	2 h	-	n.a.	2.9	76.0	6	Antioxidant activity	[50]
*Hylocereus polyrhizus* (dragon fruit)	Fruit peel	CE	C_6_H_8_O_7_	2.0	73	67 min	-	0.6	63.7	39.1	< 1	Cholesterol-regulating property	[48,75]
*Malus domestica* (apple)	Pomace	SWE	H_2_O	-	150	5 min	-	4.5	86.0	52.2	53	Antioxidant activity; antitumor activity; gel-forming property; shear-thinning property	[35]
		MAE followed by heat treatment	HCl	1.9	-	30 min	945 W	n.a.	64.8	68.5	1158	Antioxidant activity; foaming activity;	[36]
					100	1 h	-						
		UAE followed by heat treatment	HCl	1.9	-	30 min	700 W	n.a.	64.2	64.9	1158	Antioxidant activity; emulsifying activity	
					100	1 h	*-*						
		MAE	C_6_H_8_O_7_	2.0	-	10 min	450 W	n.a.	47.7	65.7	< 1	Antioxidant activity; binding and coating agent for food and pharmaceutical products	[38]
		CE	Na_2_CO_3_ + NaBH_4_	7.0	room	24 h	-	1.4	4.9	55.1	n.a.	Gel-forming property; shear-thinning property;	[37,76]
*Mangifera indica* (mango)	Fruit peel	UAE	C_6_H_8_O_7_	2.5	80	15 min	500 W	n.a.	88.6	53.3	2320	Emulsifying activity; shear-thinning property;	[62]
*Musa paradisiaca* (banana)	Fruit peel	MAE	HCl	3.0	195	60 s	1000 W	n.a.	2.0	26.0	1	Prebiotic properties; shear-thinning property; viscosity modifier for food and pharmaceutical products;	[63,77]
		UAE	H_2_SO_4_	2.5	10–15	20 min	300 W	n.a.	3.2	69.2	< 1	Emulsifying activity; shear-thinning property; viscosity modifier for food and pharmaceutical products	[61]
*Opuntia albicarpa* (pricky pear cactus)	Fruit peel	CE	EDTA	4.0	70	2 h	-	2.6	30.7	65.4	1016	Gel-forming property; shear-thinning property;	[53]
*Passiflora edulis f. flavicarpa* L. (passion fruit)	Fruit peel	UAE followed by MAE	(NH_4_)_2_SO_4_	5.0	25	30 min	400 W	0.9	65.0	68.2	363	Shear-thinning property;	[66]
						9 min	600 W						
*Passiflora tripartita* var. *mollissima* (banana passion fruit)	Fruit epicarp	CE	HCl	1.0	90	1 h	-	n.a.	52.0	82.2	14	Emulsifying activity	[45]
*Rubus chingii* Hu (blackberry)	Leaves	CE	H_2_O	-	87.9	3.1 h	-	<0.1	n.a.	16.4	17	Antitumor activity; immunomodulatory activity; antioxidant activity	[44]
*Solanum betaceum* (red chilto)	Fruit peel	CE	H_2_O	-	100	2 h	-	1.4	60.8	77.6	n.a.	Antioxidant activity; emulsifying activity; film-forming ability; foaming activity; hypoglycemic potential; inhibitory activity to α-amylase;	[41,78]

C_6_H_8_O_7_—citric acid; (NH_4_)_2_C_2_O_4_—ammonium oxalate; CE: conventional extraction technique; EAE: enzyme-assisted extraction; MAE: microwave-assisted extraction [W]; UAE: ultrasound-assisted extraction [W]; HCAE: hydrodynamic cavitation-assisted extraction [W]; UHPAE: ultra-high-pressure-assisted extraction [MPa]; SWE: subcritical water extraction; UF: ultrafiltration [Bar]; PES: polyethersulfone membrane; CEL: cellulase; hCEL: hemicellulase; PE: pectinase; PG: polygalacturonase; PL: pectate lyase; PME: pectin methyl esterase; EAR: endo-1,5-α-arabinanase; n.a.—data not available.

### 2.2. Overview of the Structural Features of Pectins Extracted from Vegetable Biomass

The pectin-like polysaccharides present in the cell walls of vegetables are slightly different from a structural point of view, especially in terms of the GalA content and DE. In general, the conventional technique for the isolation of vegetable-based pectins is hot acid extraction [23,54,79,80,81,82,83,84,85], which, in fact, is the CE method. Hot mineral acid extraction resulted in LMPs of sugar beet root [84] and potato tuber pulp [79], and HMPs of tomato waste by-products (pulp, skin, and seeds) [81] and broccoli stalks [23,83]. However, an extraction of hot organic solvents provided only the LMP of black carrot pomace [85] and roots [80], potato tubers [54], and sunflower heads and steam [82]. Extraction with water only at a temperature near the boiling point (70–100 °C) resulted in HMPs of edible burdock root [12] and sugar beet root pulp [30].

However, environmental-friendly approaches have also been introduced to some extent to vegetable waste products. The UAE process provided the HMP of the eggplant peel [86] and pumpkin fruit pulp [87], as well as the LMP of sunflower heads and stems [88]. The MAE of the calyx of the eggplant fruit resulted in HMP [89]. The enzyme-assisted extraction approach towards artichoke bracts, leaves, and stems resulted in LMPs. Song et al. [90] performed the enzymatic extraction of pectins from leaves and obtained branched pectins only with different HG/RG-I rations and moderate GalA content (~30%), depending on the type of enzyme used. Furthermore, a combined approach of ultrasounds, alkaline medium (pH~13.0), organic solvent, and cellulase applied to maize husks resulted in an LMP of high GalA content [91].

To summarize the influence of extraction conditions on the properties of pectins isolated from vegetable waste through CE, the following dependencies were observed: (i) hot water extraction favors HMPs of moderate-to-high GalA content (40–69%) and Mw > 6 kDa [12,30]; (ii) hot mineral acid extraction favors pectins of HG > RG-I, high DE (56–77%), high GalA content (74–80%), and Mw > 72 kDa [23,81,83], or pectins of HG < RG-I, low DE (18–29%), moderate GalA content (29–52%), and Mw > 280 kDa [79,84]; (iii) hot organic solvent extraction favors pectins of HG > RG-I, moderate DE (18–36%), high GalA content (58–82%), and Mw > 597 kDa [54,80,82]. Due to the fact that vegetable waste by-products are often overlooked as a source of pectins, there are few reports in the literature on the modification of CE toward the development of those that are less harmful to the environment EPT. However, several relationships can be observed: (i) UAE favors pectins of HG > RG-I, high DE (61–72%), high GalA content (> 60%) and Mw > 26 kDa [86,87], or pectins of HG > RG-I, low DE (8–34%), high GalA content (67–73%), and Mw = 108–175 kDa [88,91]; (ii) EAE favors pectins of HG > RG-I, moderate GalA content (28–32%), and Mw = 14–79 kDa [90].

**Table 2:** Overview of extraction conditions, main structural features, and functionality of pectins from selected waste and by-products of vegetables.

Plant (Vegetable)	Waste By-Product	Extraction Technique	Conditions of Extraction					$\frac{HG}{RGI}$	DE (%)	GalA (%)	Mw (kDa)	Utility	Ref.
			Medium	pH	T (°C)	t	Factor						
*Arctium lappa* L. (edible burdock)	Root	CE	H_2_O	-	85	3 h	-	0.6	67.5	68.8	1840	Anti-constipation activity; Shear-thinning property	[12]
*Beta vulgaris* (sugar beet)	Root pulp	CE	H_2_O	1.8	85	30 min	-	2.9	60.6	40.8	6–82	Prebiotic properties	[30]
	Root	CE followed by alkali treatment	HNO_3_	1.7	70	4 h	-	0.6	18.0	52.5	419	Antitumor activity	[84]
*Brassica oleracea* var. *Italica* (broccoli)	Stalk	CE	HNO_3_	2.0	100	30 min	-	2.7	56.2	74.7	72	Immunomodulatory activity; emulsifying activity; foaming ability; shear-thinning property;	[23,83]
*Cucurbita maxima* (pumpkin)	Fruit pulp	HCAE	H_2_O	3.7–4.2	65–70	15–30 min	-	1.6	71.9	62.0	26–96	Antioxidant activity; cytoprotective effect	[87]
*Cynara scolymus* L. (artichoke)	Bracts, leaves, and stems	EAE	H_2_O	5.0	50	48 h	CEL	3.7	19.5	75.7	5–660	Immunomodulatory activity	[92,93,94]
*Daucus carota* L. *Ssp. Sativus* var. *Atrorubens* Alef. (black carrot)	Pomace	MAE	CH_3_COOH	2.5	110	5 min	180 W	n.a.	38.3	32.8	1170 *	Antioxidant activity	[85]
	Root powder	UAE & EAE	Na_3_C_6_H_5_O_7_	5.2	-	20 min	600 W + hCEL	1.2	42.0	50.0	35–787	Antioxidant activity; emulsifying activity; film-forming property; gel-forming property	[80,95]
*Nelumbo nucifera* Gaertn (lotus)	Leaves	EAE	H_2_O	4.5	50	48 h	AMS	0.6	n. a.	32.0	79 16	Immunomodulatory activity	[90]
		EAE	H_2_O	4.5	50	48 h	CEL	0.3	n.a.	31.0	16	Immunomodulatory activity	
		EAE	H_2_O	4.5	50	48 h	PE	0.1	n.a.	28.7	15	Immunomodulatory activity	
*Solanum lycopersicum* (tomato)	Fruit skin, seed, and pulp	CE	HCl	2.0	85	1 h	-	n.a.	76.3	80.0	n.a.	Gel-forming property; Shear-thinning property;	[81]
*Solanum melongena* (eggplant)	Fruit peel	UAE	C_6_H_8_O_7_	1.5	-	30 min	50 W	n.a.	61.2	66.1	n.a.	Antioxidant activity; Emulsifying activity; foaming capacity	[86]
	Fruit calyx	MAE	C_6_H_8_O_7_	1.5	-	2 min	700 W	1.3	60.7	60.2	n.a.	Antioxidant activity; emulsifying activity; foaming capacity	[89]
*Solanum tuberosum* L. (potato)	Tuber peel	CE	C_2_H_2_O_4_	4.6	85	2 h	-	1.3	35.9	58.4	1819	Emulsifying activity; shear-thinning property	[54]
		CE followed by HPH	C_2_H_2_O_4_	4.6	85	2 h	-	1.1	18.0	72.3	597	Emulsifying activity	
			C_2_H_2_O_4_	4.6	-	5 min	200 MPa						
	Tuber pulp	CE	HCl	2.0	90	1 h	-	0.5	28.6	29.8	280	Emulsifying activity	[79]
*Zea mays* (maize)	Husks	UA pretreatment followed by NaOH and EAE	H_2_O	-	-	20 min	750 W	5.2	8.8	67.0	109	Gel-forming property; texture modifiers for food and pharmaceutical products	[91]
			NaOH	13.0	-	-	-						
			Na_3_C_6_H_5_O_7_	5.2	40	4 h	CEL						
*Badami cultivar* (sunflower)	Heads and stems	UAE	H_2_O	-	33	30 min	400 W	3.0	34.1	72.9	175	Antioxidant activity; emulsifying activity; foaming activity	[88]
		CE	(NH_4_)_2_C_2_O_4_	-	85	45 min	-	2.8	27.3	82.1	606	Shear-thinning property	[82]

CH_3_COOH—acetic acid; C_2_H_2_O_4_—oxalic acid; C_6_H_8_O_7_—citric acid; Na_3_C_6_H_5_O_7_—sodium citrate; CE: conventional extraction technique; EAE: enzyme-assisted extraction; MAE: microwave-assisted extraction [W]; UAE: ultrasound-assisted extraction [W]; HCAE: hydrodynamic cavitation-assisted extraction [W]; HPH: high-pressure homogenization [MPa]; CEL: cellulase; hCEL: hemicellulase; PE: pectinase; AMS: α-amylase; *: particles size (nm); n.a.—data not available.

### 2.3. Overview of the Structural Features of Pectins Extracted from Miscellaneous Plant Biomass

The interesting group of food waste by-products is inedible parts of nuts, herbs, and beans, such as pods, husks, hulls, needle-shaped leaves, roots, etc. The isolation of pectins from this type of biomass is usually a more complex process than in the case of fruit or vegetable biomass. This is due to the higher content of hemicelluloses and lignin that makes the cell wall more difficult to disrupt and penetrate to extract pectins [96].

By far, the most efficient pectin-separation technique for this type of biomass appears to be the conventional hot-medium approach. Hot water extraction provided RG-I-dominated LMPs from buttonwood leaves [97]. Hot mineral acid extraction resulted in moderately branched LMPs of the cocoa pod husk [98], while hot organic acid extraction resulted in moderately branched LMPs of shrubby seablite leaves [99] and cocoa pod husk [100,101]. EPTs (that is: mineral acid-UAE, mineral acid-MAE, enzyme-assisted) were used to extract the HMP from walnut green husks [102,103], as well as LMPs from pistachio hulls [104,105] and cocoa pod husks [106]. The common feature of this particular type of biomass is that it provides mostly moderately branched LMPs with a high GalA content (>59%). Moreover, the structural diversity of pectins derived from miscellaneous waste biomass depends on the separation technique to a much lesser extent than in the case of pectins derived from fruit and vegetable waste by-products.

**Table 3:** Overview of extraction conditions, main structural features, and functionality of pectins from selected waste and by-products of nuts and leaves.

Plant (Nuts)	Waste By-Product	Extraction Technique	Extraction Parameters					$\frac{HG}{RGI}$	DE (%)	GalA (%)	Mw (kDa)	Utility	Ref.
			Medium	pH	T (°C)	t	P (W)						
*Conocarpus erectus* (buttonwood)	Leaves	CE	H_2_O	-	60	4 h	-	0.4	37.5	36.0	24	Antioxidant activity; immunomodulatory activity; prebiotic effect;	[97]
*Juglans regia* L. (walnut)	Green husks	UAE	HCl	1.5	-	10 min	200 W	n. a.	59.2	69.4	93	Antioxidant activity; emulsifying activity;	[103]
		MAE	HCl	1.5	-	3 min	500 W	n.a.	54.1	68.4	260	Antioxidant activity; emulsifying activity;	[102]
*Nicotiana tabacum* L. (tabbaco)	Root	UAE followed by EAE	(NH_4_)_2_SO_4_	-	-	9 min	180 W	0.1	n. a.	2.3	n.a.	Antioxidant activity	[107]
			H_2_O	-	50 °C	1.5 h	CEL PE						
*Pistacia vera L.* (pistachio)	Hull	UAE	H_2_SO_4_	1.5	-	24 min	150 W	n.a.	41.3	59.3	n.a.	Antioxidant activity; emulsifying activity; foaming activity;	[104]
		MAE	H_2_SO_4_	1.5	-	165 s	700 W	1.8	12.1	66.0	2	Antioxidant activity; emulsifying activity;	[105]
*Suaeda fruticose* (shrubby seablite)	Leaves	CE	C_6_H_8_O_7_	2.9	90	37 min	-	0.9	33.0	47.5	229	Analgesic properties; immunomodulatory activity; antioxidant activity;	[99]
*Theobroma cacao* L. (cocoa tree)	Pod husk	CE	HNO_3_	3.5	100	30 min		1.1	41.0	59.2	1989 229	Gel-forming property; shear-thinning property;	[98]
		CE followed by saponification	C_6_H_8_O_7_	3.0	95	1.5 h	-	0.7	20.8	56.0	259	Antimicrobial activity; immunomodulatory activity;	[101]
			NaOH + NaBH_4_	-	4	16 h	-						
		CE	C_6_H_8_C_6_	2.5	95	45 min	-	n.a.	8.1	74.5	n.a.	Shear-thinning property;	[100]
		EAE	Na_3_C_6_H_5_O_7_	4.6	50	18.6 h	CEL	n.a.	24.0	52.1	n.a.	Gel-forming property; shear-thinning property	[106]

C_6_H_8_O_7_—citric acid; C_6_H_8_O_6_—ascorbic acid; Na_3_C_6_H_5_O_7_—sodium citrate CE: conventional extraction technique; EAE: enzyme-assisted extraction; MAE: microwave-assisted extraction [W]; UAE: ultrasound-assisted extraction [W]; CEL: cellulase; PE: pectinase; n.a.—data not available.

## 3. Techno-Functional Usefulness of Pectins

Differences in the structure of pectin derived from a particular plant material are obviously affected by the extraction conditions and purification procedure. However, the enzymatic activity that corresponds to the ripening stage of the plant is often negligible [26]; that is, molecular weight and monosaccharide composition are influenced by the activity of polygalacturonase or pectate lyase [108], while the degree of esterification is the result of the activity of pectin methylesterase [26]. The diversity of the structure of these compounds also translates into their potential utility. Generally, the techno-functional properties of pectin vary over a wide range depending on the source of extraction. Because most of these compounds are biodegradable and safe to be in contact with the human body, they have the potential to be used in food and pharmaceutical products, depending on their physicochemical properties for multi-target beneficial effects. The large amount of waste by-products from the food industry could be recovered as a source of valuable biopolymers immediately after the cultivation period even without the prior drying process [23].

### 3.1. Structure–Function Relationship of Pectin at Interfaces

Emulsions are composed of at least two phases forming a macroscopically homogeneous system, i.e., immiscible liquids, liquid and gas, or liquid and a solid, where one is finely dispersed in the other one. The fundamental components of these systems are surface-active agents that reduce interfacial tension and thus stabilize dispersions. Most surfactants are amphiphiles and may interact with both phases of dispersed systems, usually via polar–nonpolar interplay. Often, these substances remain ionized in an aqueous environment and aid in system stability with electrostatic and steric interactions as well [109]. Pectin is abundant in carboxylic moieties that are ionized under specific conditions (pH < 3.5) and contribute to stabilizing colloids with non-esterified galacturonic acid moieties that ensure a widely distributed charge net. The relatively high degree of methylation might reflect its amphiphilic character and suggests that it might adsorb at the interface of immiscible phases, i.e., liquid–air and liquid–liquid interfaces, and it decreases the surface tension. Otherwise, the strong hydrophilic nature of pectin impedes its surface activity. Studies on pectin derived from palmyra palm fruit pulp [42], watermelon fruit rinds (via hot-acid extraction) [26], bigarade fruit pulp [46], orange fruit peel [33], apple fruit pomace [36], banana passionfruit fruit epicarp [45], chilto fruit peel [78], and walnut green husks [102,103] revealed an acceptable emulsifying activity for HMP.

Other biopolymers, i.e., protein moieties, contribute to an amphiphilic character of pectin and enhance its emulsifying and foaming properties [102,110]. The protein component adsorbs on the surface of oil droplets, and the carbohydrate part anchors in the aqueous phase [23] and efficiently contributes to stabilizing emulsions [23]. Usually, proteins prefer to be coupled to the uncharged side chains [8]. Thus, under specific conditions (pI > pH > pKa), the attractive electrostatic interactions between (+) amine groups in proteins and (−) carboxylic groups in GalA result in tighter structure and less steric hindrance. This behavior was observed for LMP with high-protein components derived from potato tuber peel [54] and pulp [79], carrot root [80,95], banana peel [61], and even pistachio hulls [104,105]. Similar emulsion activity was reported for HMP with a high-protein component derived from mango fruit peel [62], broccoli stalks [23], eggplant fruit peel [86], and calyx [89].

### 3.2. Rheology of Pectin Solutions and Gels

Pectins, in general, are identified as applicable to control the rheology and texture of various formulations, especially food products [111]. Pectin derived from apple pomace waste is one of the best known in this regard, and, at least since the 1990s, the functional properties of these substances have been widely studied [111,112]. One of the most essential functional properties evaluated for pectin for use in food and pharmaceutical products is the flow behavior. Most pectins modify the viscosity of aqueous solutions similarly to other hydrocolloids, and, generally, an increase in viscosity is associated with strong shear-thinning behavior at low concentrations [110]. The extension of this phenomenon could be assigned to the disentanglement of the polymer network from intermolecular and intramolecular interactions and the partial orientation of the chain in the shear flow direction with an increasing shear rate [102,109]. Some pectin in low concentrations imparts properties similar to Newtonian fluids when its viscosity is unaltered regardless of the shear force. This is probably an effect of weak pectin–pectin interactions because the rate of disruption of physical entanglements between chains is slower than the rate of formation of new ones, especially for branched structures. The shear-thinning characteristic becomes more pronounced with increasing concentration due to the predominant disruption of the pectin network [53,102]. The Mw content, the GalA content, the DE, and the type of neutral monosaccharides and their conformation obviously directly affect the properties of pectin in solutions with regard to viscosity. The lower the molecular weight of pectin, the more shear-thinning fluid flow is observed under the influence of the shear force and provides a less thickening effect, i.e., higher pseudoplasticity [110]. Longer pectin chains provide more viscous solutions due to a stronger coherent network and more rigid structure. Therefore, it is more difficult for chains to orient consistently with the direction of the shear flow [111]. DE also affects the thickening properties of pectin as a result of the hydrophobic nature of the methoxy groups. The viscosity of aqueous solutions of pectin usually decreases with the lower amount of methylated carboxyl groups. The more GalA units, the more pectin that is abundant in highly accessible hydroxy groups, resulting in more water molecules being trapped within the pectin structure and pectin–pectin hydrogen interactions, resulting in enhanced viscosity. Along with an increase in the amount of methoxy moieties, pectin is less hydrated and provides less viscosity to the solution. The contribution to viscosity in pH < 3.5 solutions of pH < 3.5 is also lower because the ability of protonated carboxylic groups in GalA to form hydrogen bonds with water molecules is limited [113]. These were confirmed by comparing the viscosities of HMP from broccoli stalks (η~100 Pas) [23] and HMP from watermelon rinds (η~32 Pas) [26] studied under the same conditions. The difference is mainly attributed to different molecular masses (72 kDa versus 31 kDa) and DE (56% versus 63%). HMP was reported to have a time-independent shear-thinning behavior from kiwi fruit pomace [22], jackfruit peel waste [25], watermelon rinds [26], mandarin fruit peel [35], apple fruit pomace [35], bigarade fruit peel [46], grapefruit peels [56], mango fruit peel [62], passion fruit peel [66], tomato processing waste [81], broccoli stalks [23], and edible burdock root [12]. Although the general recommendation for food-grade pectins of GalA is higher than 65%, some LMPs with a GalA content <65% may also be interesting due to the shear characteristic. The shear-thinning properties of LMP are from apple pomace (alkali-soluble) [37], kinnow fruit peel [49], banana fruit peel [61,63], prickly pear cactus fruit peel [53], potato tuber peel [54], sunflower heads and stems [82], and cocoa pods [98,100,106]. The protein or polyphenol component can provide a steric contribution that increases the viscosity of the pectate system [114]. The hydrophobic interactions observed between aromatic rings of polyphenols and hydrophobic methyl groups of pectin are considered the primary mechanism of polyphenol–pectin complexation [47]. These waste-derived pectins are promising candidates for texture modifiers in the food and pharmaceutical sectors that require shear-thinning properties due to technological requirements (pumps, mixing, packaging, and transport) and pleasant mouthfeel.

Typically, polysaccharides in aqueous solutions at low oscillatory frequencies are characterized by a lower storage modulus (G′) (so-called elastic modulus) than the frequency loss modulus (G″) (so-called viscous modulus), indicating the dominance of viscous properties of the chain due to dynamic equilibrium between the pectin molecular net and the shear force. Often, this frequency range is rather narrow, below 10 Hz, but sometimes even < 1 Hz. When frequency increases, the opposite event occurs, i.e., G′ > G″, suggesting that pectin exerts an elastic character, or that gel formation begins due to the steady orientation of the pectin chains and interchain association [35,115]. The value of the crossover frequency (CF) between G′ and G″ indicates exactly when the viscoelastic transformation occurs for a pectin. The lower the crossover frequency, the more pronounced the elastic contribution. The mechanical response of pectin depends on Mw, DE, and the ability of the chains to orient consistently and exhibit elastic properties; that is, pectin of low Mw can easily orient consistently [25]. Moreover, other components of pectin conjugates probably also affect the viscoelastic behavior of pectin, especially if electrostatic interactions occur, meaning negatively charged GalA and positively charged side chains of proteins [35]. The dynamic viscoelastic behavior evaluated for HMP derived from jackfruit peel waste [25], watermelon rinds [26], mandarin fruit peel [35], apple fruit pomace [35], passion fruit peel [66], edible burdock root [12], broccoli stalks [26], and tomato processing waste [81] revealed that all the tested pectins displayed relatively strong elastic characteristics G′ > G″. A similar characteristic for LMP was reported for the kinnow fruit peel [49], melon fruit peel [51], and the cocoa husk pods [98], implying that the LMP was more elastic than viscous. The LMP of the potato tuber peel did not exhibit viscous behavior but was only elastic within oscillatory measurements frequency sweep tests 1–10 Hz [54]. Lira–Ortiz studied Ca^2+^-induced gelation of LMP from prickly pear cactus fruit peel [53] and denoted a gel-like behavior of LMP with a positive correlation with increased concentration of a cross-linking agent, resulting in soft and elastic gels. The opposite mechanical response was reported for LMP from grapefruit peel [56] that presented liquid-like behavior. Wang et al. [56] linked this behavior to the high percentage of side chains attached in HG-I. From the techno-functional applicability of pectin, if the elastic properties are higher than the viscosity, this may suggest that pectin is more applicable as a texture modifier in the final products rather than as a thickener, especially if pectin exerts shear-thickening behavior [35].

Nearly all types of pectin are capable of forming gels under strictly defined conditions. The most versatile are LMPs in this respect. LMPs form gels through electrostatic interactions between divalent ions, such as Ca^2+^, Mg^2+^, and Fe^2+^, as well as charged carboxyl groups of smooth homogalacturonan regions (HG) under pH > 4.5; that is, gelation follows the egg-box model. The stability of the gel depends on neighboring non-esterified GalA residues as they form a junction zone for a divalent cation. LMP gels are also stabilized by van der Waals forces between adjacent pectic chains and hydrogen bonds between polar groups of pectin molecules [98]. It is not surprising that fruit waste is abundant in LMP that is capable of forming stable gels with divalent ions, i.e., true gels. Vriesmann et al. received calcium–pectate gel composed of LMP derived from cacao pod husk [98], Gawkowska et al. [37,76] studied zinc–pectate gel composed extremally low methylated pectin (DE = 4.9%) from apple fruit pulp extracted under alkaline conditions, Lira–Ortez et al. [53] received a calcium–pectate gel based on LMP polysaccharide form prickly pear cactus fruit peel. However, Idrovo et al. [80] received a stable calcium pectate gel from LMP extracted from carrot root pomace, and Higuera–Coelho et al. [91] received stable calcium pectate gel and a weak iron (II) pectate gel with LMP from maize husks. Moreover, to form a stable gel, non-methyl esterified GalA moieties are required to be organized in ca. 6–20 units along the pectin chain. A high proportion of hydrophobic acetyl groups and inserts of rhamnogalacturonan I (RG-I) side chains hinder chain–chain association and impair gel formation as a consequence of steric disorders [98]. LMPs are capable of gel formation even without divalent cations, but then the mechanism follows pectin–pectin interactions by hydrogen bonds. This is possible only if the electrostatic repulsions between the GalA groups are marginal; that is, pH < 3.5, and water activity is low [98]. This behavior was observed for LMPs derived from cocoa tree husk pods [106] and chocolate wine fruit peel, with the exception that the latter LMP formed soft gels only under pH = 2.0 [52].

On the other hand, HMPs undergo sugar–acid-mediated gelation. Such a colloid is stabilized by non-covalent bonds of adjacent chains in junction zones, i.e., hydrogen bonds between galacturonans and hydrophobic interactions between esterified groups were assisted by a high co-solute (glucose) concentration (7–60%) due to reduced water activity. Moreover, high DE promotes attractive forces such as the van der Waals’ interactions that contribute to the gelation process as well. Altogether, these interactions result in a tightly entangled network stable in acidic conditions [35,42,114]. If the pH increases > 3.5, the HMP gels become weaker due to electrostatic repulsions arising from the gradual deprotonation of the carboxyl groups [114]. HMPs derived from jackfruit peel [25] and HMPs from Palmyra palm fruit pulp [42] formed sugar–acid pectin gel regardless of pectin composition. However, gels became softer as the number of methylated carboxyl groups in the structure increased. The results of calcium pectate hydrogels composed of HMPs from lemon fruit peel revealed deacetylation sensitizes HMP to cross-linking with Ca^2+^ in terms of gel development [29,33]. The HMP from tomato processing waste was derived via acidic extraction, followed a two-step gelation mechanism: at high temperatures, it was driven by hydrophobic interactions, while at lower temperatures, it was driven by hydrogen bonds [81]. Wang et al. [35] compared the final properties of sugar–acid pectin gels composed of HMP derived from mandarin fruit peel with the one composed of HMP derived from apple pomace. Since citrus HMP produced a stronger gel than apple HMP, it was proved that the hardness and elasticity of pectin gel depend not only on DE but also on Mw profoundly impacting the gelling properties of pectins. Li et al. [25] and Begum et al. [24], independently of each other, reported similar findings for jackfruit HMP-based gels—the strength of pectate gels increased along with higher molecular weight due to an increase in the number of interactions between pectin chains.

Other techno-functional properties such as the water-holding capacity (WHC) (that is, the amount of water retained per 1 g of pectin) and the oil-holding capacity (OHC) (that is, the amount of oil retained per 1 g of pectin) are greatly affected by the total charge density and the hydrophobic nature of pectin, which in fact is a result of the chemical composition, structure, and pH of pectin [116]. The WHC of pectins is usually influenced by the GalA content, the number of free hydroxyl groups, the size of the particles, and the molecular structure. The influence of various factors on the OHC parameter may include porosity, hydrophilic nature, and the overall charge density of the surface [46]. El Fihry et al. [46] state that the higher the number of free hydroxyl groups in the HG region, the lower the DE, which increases the ability of pectin to bind more water resulting in better WHC. The HMPs of the bigarade fruit peel [46], the chilto fruit peel [78], eggplant fruit peel and calyx [86,89], and the green walnut husks [103] exert greater water than the oil-holding capacity. A similar trend was observed for the LMP of kinnow fruit peel [8], sunflower heads and stems [88], and pistachio hulls [104,105]. The opposite characteristic was observed for the HMP of apple pomace [36]. When GalA, DE, and Mw are considered for pectins with different characteristics, it appears that the OHC and WHC parameters are not related to each other. However, it may be assumed that WHC is related to the amount of GalA in the structure. A high WHC parameter contributes to improving the textural properties, modifying the viscosity, and reducing the number of syneresis incidents in the final products. On the other hand, the high OHC parameter contributes to the dispersion of immiscible liquids, indicating that pectin can serve as a stabilizer of the oil phase in emulsions or final products with high fat content [36,78,89].

### 3.3. Pectins as Functional Biomaterials

By-products of the food industry provide pectins ready for use as biomaterials for active and/or edible films, hydrogels, or microstructures, such as beads, for food and pharmaceutical applications. Pectin provides flexible films, which are resistant to handling and have a smooth surface, regardless of the concentration of the pectin used. The films effectively retain water due to the hydrophilic character of pectin, and most of them exert good water vapor permeability. The mechanics behind film formation are that the GalA content is the main factor that influences the mechanical strength and stiffness of pectin-based films [47]. Moreover, the higher the galacturonic acid content and the degree of esterification, the higher the effects of the moisture and oxygen barrier of the film. However, the increase in the degree of acetylation is correlated with an increase in hydrophobicity because hydrophobic acetyl groups replace hydrophilic ones [47]. The possibility of formulating a composite film of waste pectins blended with one or more other polymers into the film matrix expands the possibilities of application as nontoxic, biocompatible materials. The protein component distributed within the pectin film matrix disturbs the homogalacturonan film, i.e., the entangled linear pectin network, and makes the film surface rough. Moreover, the more protein components that contribute to the film, the more spherical formations that might be formed by tiny protein-stabilized air bubbles [47]. The protein component increases the effect of the moisture barrier, in contrast to the phenolic component. The final mechanical, barrier, and surface properties of pectin-based films are affected by the internal film morphology and the molecular and compositional parameters.

LMPs from apple pomace were used as a binding, coating, and protective agent for dried fruit bars [38]. The edible apple pectin film, applied on the surface of a food product, contributed to reducing moisture loss, minimizing the degradation of bioactive compounds, decreasing gas exchange, and maintaining the potential antioxidant activity of the product during storage [38]. The pectin film on the surface of the bars reduced interactions between the other components of the bars and the environment and preserved its nutritional value for more than a year [38]. The LMP of pineapple peels has been explored to be an efficient plasticizer in edible films [64]. The properties of the films composed of purified pineapple pectin were not acceptable, therefore, the raw extract containing pectin and phenolic compounds was applied to produce films. The properties of the final films were obviously affected by the phenol component but were still of high usability. For pectins coupled with polyphenols, the moisture-retention property was slightly limited because the polyphenol compound limits the bonding of hydrogen groups with water. This also influenced the water vapor permeability of pectin films because the covalent interactions between pectin chains and phenolic compounds limit the availability of hydrogen groups to form hydrophilic bonds with water molecules [64]. The outcome was visible in the film macrostructure that shifted from homogeneous to heterogeneous with local disturbance of the usually smooth surface of the pectin films. Furthermore, the phenolic component resulted in a more rigid and less extensible film compared to the pure pectin film [64]. The LMP from black carrot root pomace occurred to form a homogenous film stabilized with Ca^2+^ and glycerol as a plasticizer [95]. Along with the pectin, some carotenoids and tocopherol were co-extracted, which are probably responsible for the water resistance of the films and limited moisture content. The film was less resistant to elongation at rupture and more flexible than the film produced with commercial pectin. This led to the application of the film as an active barrier from lipid peroxidation in a cashew-ripened cheese for at least 60 days. Moreover, the film stabilized the orange color of the food product even during storage under room conditions [95]. Composite films prepared from blood orange peel HMP and fish gelatin were developed as an active packaging material for Ricotta cheese to extend its shelf life; that is, to improve the physicochemical and textural properties, as well as the microbial stability of cheese during chilled storage [32]. The edible films increased the physicochemical and nutritional values of the wrapped cheese. The HMP did not affect the UV light barrier of gelatin, and protection from oxidative deterioration and loss of nutrients was maintained for the composite films. The transparency of the film was slightly reduced due to polyion complex interactions between both biopolymers within the film formation network, but this is not surprising as this parameter is determined by morphology rather than chemical composition [47]. However, HMP contributes to the clear yellow color of wrapped cheese over the storage period compared to gelatin-based film and unwrapped pieces. The composite film acted as a semi-permeable barrier that reduced water loss over time. This allowed it to maintain the low hardness of the food product and increase its cohesiveness and chewiness [32]. Çavdaroğlu et al. [47] developed edible films composed of HMP from fig fruit stalks and cross-linked with Ca^2+^. These films are interesting because pectin-based functional materials derived from waste and stabilized via safe cross-linking agents are limited. Despite the fact that the cross-linking agent interacted only with the LMP fraction of the fig stalk pectin, this significantly altered the final properties of the films. In comparison to films formed with HMP from fig fruit stalks without a cross-linking agent (pristine films), the cross-linked films were ca. 10% thinner, showed ca. 20% higher tensile strength, were twice as flexible, were ca. 35% more stiffness, and were more hydrophobic [47]. The cross-linked film exhibited a great moisture and oxygen–gas barrier effect because of the high degree of acetylation and dense morphology. Results discussed by Çavdaroğlu et al. [47] suggest that the cross-linking caused the formation of denser morphologies for fig pectin films and the high degree of acetylation interfered with the gelation of pectins since the presence of acetyl groups caused steric hindrance for chain association. The cross-linked film formed an extensive tiny aggregation visualized within the films [47]. HMP extracts of red chilto fruit peel were applied as a matrix in a biopolymer film to entrap the polyphenolic-enriched and anthocyanin-enriched [41]. The edible films showed a high antioxidant capacity and good mechanical and barrier properties, and they protected salmon filets from oxidation and extended their shelf life [41]. Active, edible films based on waste by-product pectins are an interesting alternative for commercial non-biodegradable synthetic food-coating materials.

Yu et al. [52] used LMPs from chocolate wine fruit peels as a biological AgNO_3_ reductant to colloidal Ag under alkaline conditions, via carboxyl groups and unreacted hydroxyl groups in the pectin chain. Wine fruit pectin stabilized, controlled the size distribution, and prevented the aggregation of silver nanoparticles [52]. Moreover, wine fruit pectin provided rapid surface wettability, good water absorption, and long-term water retention, which resulted in the ability of pectin-based sponges to maintain a moist environment underneath [52]. In this regard, chocolate wine fruit peel LMP facilitated the healing of infected wounds in rats when used as a component of sponge-like biomaterial with silver nanoparticles [52].

Safe and efficient hydrogel-delivery systems are currently an emerging area in the food and pharmaceutical industries. For these purposes, pectins seem to be interesting candidates for building blocks for numerous types of delivery systems. The waste by-product pectins are easily available and economically reasonable. The diversity of pectins from these sources allows for the production of delivery systems of tunable morphology and properties, including homogeneous structure, size, interconnected pores, and surface area [27]. Biopolymer-based hydrogels, including food-grade polysaccharides and proteins, are still being investigated by detailed feasibility studies due to a number of factors, including the type of fruit and/or vegetable waste and its seasonality, followed by the extraction process, which obviously influences the chemical composition of the pectins received [27]. These factors affect the esterification degree, molecular weight, linearity, and even purity of pectin. Nevertheless, waste pectin-based delivery systems are considered promising carriers of active compounds due to their biocompatibility and potential benefits as a prebiotic compound. The application limitations such as fast drug release need to be addressed with some alternative strategies for release control. However, owing to the protonation/deprotonation interplay of carboxyl groups of pec-tins, including by-products waste pectins, it is possible to control the release rate with environment alterations. Those carboxyl groups that are not interacting with the stabilizing agent, become deprotonated under pH > 3.5 and electrostatic repulsions increase the intermolecular distance between pectic backbones, resulting in the hydrogel structure becoming less entangled and pore sizes and particle diameter increasing [117,118]. Moreover, pectin solutions of lower viscosity will result in microparticles of lower diameter [67]. Considering the above aspects, Méndez et al. [27] conducted very extensive studies on the suitability of LMP watermelon fruit rinds for creating aerogel microspheres. They confirmed that the lower the DE content and the higher the HG content, the stronger the calcium–pectate hydrogel. This suggests that, for a lower degree of branching, pectin forms stronger calcium-stabilized hydrogels. The structural integrity of the microbeads was affected by the biopolymer properties (concentration, composition, DE, Mw, and molecular interactions), and surface tension effects at the gas-solvent interfaces. Watermelon rind pectin provided stable hydrogel microbeads resistant to shrinkage when replacing water with ethanol and, finally, with scCO_2_ and vanillin. These results indicate that, for hydrogels of low pectin concentration, most carboxyl groups are engaged in egg-box junction zones, providing relatively hydrophobic inner sacks. For hydrogels composed of high concentrations of pectin, carboxyl groups are only partially involved in calcium interactions, providing a rather hydrophilic environment [27]. Therefore, a greater hydrophobic payload-loading capacity was observed for aerogels with lower pectin concentrations. LMPs from the yuzu fruit peel and oligochitosan were used to produce calcium-stabilized hydrogel beads for the oral delivery of quercetin targeted to the colon [67]. The addition of oligochitosan to this system resulted in electrostatic interactions between these polyelectrolytes that limited premature payload leakage from the carriers. Only a negligible amount of quercetin was shown to be released under unfavorable conditions due to the synergy of Ca^2+^ cross-links and the polyelectrolyte complex. The yuzu pectin-based hydrogels protected the payload during its stay in the upper part of the gastrointestinal tract in vitro. The beads were subjected to total hydrolysis only when treated with pectinase [67].

Dominiak et al. confirmed that HMPs from lemon fruit peel stabilized mild drinks due to very high DE levels (~80%) and Mw (225.5 kDa) [29]. In this particular case, stabilization occurred due to the common influence of attractive electrostatic interactions between HMP homogalacturonan domains and casein, and repulsion interactions between deprotonated carboxyl rests in GalA moieties surrounding casein micelles. On the other hand, extremely low methylated pectin from banana peel (DE = 2.8%) exhibited shear-thinning properties for whey protein isolate solutions [63] with comparable efficacy are exerted by guar gum [119] or carboxymethyl cellulose [120]. It also stabilized the formulation and protected it from wheying-off [77]. Moreover, banana peel LMP maintained acceptable viscosity of the formulation after freezing and thawing and during excessive storage, and it compensated for the unbalanced whey protein-to-casein ratio, or the mishandling of the product during storage and distribution [61,77]. Finally, this LMP provided an appropriate texture for orange juice under digestion conditions in vitro [63]. In this application, the formulation is stabilized via binding pectin molecules in a 3D network capable of interacting with other components of the matrix.

### 3.4. Pectins for Biological Application

Currently, pectins are extensively exploited not only as formulation agents but also as health-favorable compounds. A multitude of studies on biological properties explore the potency of them as effective biologically active biomaterials, such as films, coatings, and scaffolds, for food, nutraceutical, biomedical, and healthcare applications.

#### 3.4.1. Antioxidant Activity of Pectins

Although the mechanism of free-radical scavenging of polysaccharides is not yet fully understood, it can be assumed that the antioxidant activity of pectins is the result of the chemical composition, Mw, and the content of GalA moieties and unmethylated acidic groups. The antioxidant ability of pectins is associated with the presence of GalA moieties in their structure with active carboxyl or hydroxyl groups capable of donating proton and/or electron transfer to scavenge free radicals [21,60]; that is, the higher the GalA content, the higher the antiradical activity. Additionally, pectins with more RG-I regions with higher branching degrees and longer side chains may potentially be better antioxidants [87]. Asgari et al. [103] reported that low Mw pectins are more active antioxidants, while Ro et al. [121] suggested that in solutions with high viscosity, pectin chains have a limited ability to facilitate the interaction between GalA hydroxyl groups and free radicals. Furthermore, this activity is often enhanced with a nonpectic component conjugated with the polysaccharide, i.e., phenolics, acylated anthocyanins, or proteins, and this is directly related to the extraction method [85,97]. Phenolics often coexist with pectins via hydrogen and hydrophobic interactions, while proteins form *N*-glycosidic bonds. Such a construct does not require an exogenous antioxidant because the polyphenolic component of pectic already has this added value. The antioxidant activity of pectin films composed of LMP of pineapple peel was conformed to food simulants for aqueous and fatty foods and was positively correlated with an increase in pectin concentration [64]. LMPs of lemon peel exert antioxidant and radical scavenging activities due to the abundance of eriocitrin, neoeriocitrin, and 6,8-di-C-β-glucosyldiosmin in their composition [21,31]. LMPs of albedo grapefruit fruits were derived in the form of a complex with flavonoids and have been reported for their antioxidant effect on human neuronal cells in vitro as a result of a high naringin content [59]. Grapefruit albedo pectin preserved mitochondrial membrane potential and cell morphology in cells exposed to oxidative stress [59]. LMP dose-dependent antiradical activity was reported in vitro from the melon fruit peel [60], fig peel [65], mangosteen rind [50], black carrot pomace [85], sunflower heads and stems [88], walnut green husks [102,103], buttonwood leaves [97], shrubby seablite leaves [99], and pistachio hull (similar to ascorbic acid activity) [104,105]. Moreover, shrubby seablite pectin exerted a central antinociceptive effect on thermal stimulus in vivo in mice, which was comparable to that of tramadol and paracetamol [99]. Mzoughi et al. [99] suggested that pectin exerted its analgesic effects through supraspinal and spinal receptors [122], with a mechanism similar to opioids, that is correlated with the ability to eliminate oxygen-free radicals that increase the content of Ca^2+^ in cells responsible for inducing pain [123]. The LMP of the black carrot root pomace was co-extracted with α-carotene, β-carotene, lutein, and α-tocopherol, which contributed to the effectiveness of protecting against oxidative stress [95]. In the case of apple pomace LMP, no dose-dependent radical-neutralization activity was demonstrated in vitro, which may be related to the release of end groups of polysaccharides that are reduced due to structural modification of pectin during the extraction process conducted under long-term exposure to microwaves [38]. In this case, the relationship between antioxidant activity and polyphenol content was more pronounced. Furthermore, numerous HMPs of waste by-products derived from grapefruit peel [56], blood orange peel [32], apple pomace [36], red chilto fruit peel [41,78], pumpkin fruit pulp [87], and eggplant peel [86,89] were identified with dose-dependent antioxidant activity. Interesting findings in this regard have been reported for the HMP of the mandarin fruit peel, which turned out to be a better antioxidant than the HMP of apple pomace, despite the fact that both efficiently scavenge free radicals in a dose-dependent manner [35]. Finally, blackberry leaf pectins [44] and tobacco root pectins [107] also possess antioxidant activity despite a low GalA content (<25%).

#### 3.4.2. Immunomodulatory Properties of Pectins

Pectins are considered biological response modifiers in immune target cells because they stimulate macrophages for a direct and indirect immune response. Pectins interact with immune system cells through dectin-1 protein, mannose, and toll-like receptors that are associated with phagocytosis, macrophage activation, biochemical destruction of targeted organisms, and chemotaxis [83,124,125]. The immunomodulatory effect (anti- or pro-inflammatory) of pectins is directly related to their chemical structure and is probably associated with a high GalA content, large side chains represented by arabinogalactans, DE, and Mw < 100 kDa [92,97,126]. Amorim et al. [101] and Busato et al. [83] observed that HMPs stimulated macrophages to increase IL-10 production (anti-inflammatory cytokine) and had no effect on IL-12 and TNF-α (pro-inflammatory cytokines), while LMPs showed the opposite effect. Moreover, pectins could induce lymphocytes; that is, they activate a cascade of cellular and humoral immune responses [127]. The HMP from broccoli stalks showed a rather unusual effect in this area [83]: (i) increased peritoneal macrophage phagocytic activity, which exhibited a strong ability to recruit monocytes from blood into the peritoneal cavity and elicit these cells in mice; (ii) did not induce NO production and secretion of IL-12 and IL-1β secretion after in vitro and treatments; (iii) stimulated IL-10 production after in vivo treatment (oral application in mice); (iv) induced lymphocyte proliferation in vitro. Therefore, Busato et al. [83] suggested that broccoli pectin was absorbed by gut-associated lymphoid tissue to modulate the immune system for an anti-inflammatory response [83]. On the other hand, the grapefruit albedo LMP–flavonoid complex inhibited the neuroinflammatory response and activation of basal microglia (resident immune cells of the brain) by downregulating the phosphoinositide 3-kinase (PI3K) signaling, decreased the activation of the factor κ-light-chain-enhancer of activated B cells (NF-kB)), and suppressed the expression of the inducible nitric oxide synthase (iNOS) cascade but did not induce IL-1β and IL-6 expression [58]. Grapefruit pectin was derived from plant tissues with accompanying flavonoids and terpenes that enhance anti-inflammatory activity [58]. LMP from processing wastewater (HClaq) discharged from the satsuma mandarin canning process significantly decreased the levels of serum TNF-α in obese mice [73]. In in vivo studies in colitis mice, LMPs of artichoke by-products showed an anti-inflammatory potential, which was greater than that of citrus HMP (positive control). Artichoke pectin reduced the expression of TNF-α, IL-1β, IL-6 cytokines, and pro-inflammatory markers iNOS and ICAM-I in a dose-dependent manner [92]. Sabater et al. [92] suggested that LMP interacts with TLR-2 through electrostatic forces between nonesterified GalA moieties and TLR-2 ectodomains, thus inhibiting the pro-inflammatory TLR-2–TLR-1 pathway. Other in vivo studies in rats revealed long-lasting and dose-dependent anti-inflammatory efficacy of LMP from shrubby seablite leaves, comparable to diclofenac used as a reference drug [99]. Shrubby seablite pectin inhibited the release and/or antagonized action of inflammatory mediators (histamine, serotonin, and prostaglandin E2) and decreased the level of NO [99]. Furthermore, the peripheral antinociceptive capacity of shrubby seablite pectin in vivo in mice was higher than the activity of the reference drug; that is, acetylsalicylate of lysine [99]. The antinociceptive action of pectin could be ascribed to the inhibition of pain-induced release of bradykinin, histamine, prostaglandin E2, cytokines (TNF-α, IL-1β, and IL-8), and substance P [99]. LMP from buttonwood leaves exerted an immunomodulatory effect; that is, it mediated innate immunity by promoting the activation and proliferation of TCD4+ lymphocytes, stimulating the secretion of pro-inflammatory cytokines (IL-2, IL-4, IL-6, IL-10, TNF-α, IFN-γ), and inducing phagocytic activity [97]. The LMP of the cocoa pod husks promoted an expressive increase in anti-inflammatory (IL-10) and pro-inflammatory (TNF-α and IL-12) cytokines and increased the level of NO in murine peritoneal macrophages in vitro [101]. In this sense, cocoa pectin activated macrophages to a cytotoxic phenotype and induced a pro-inflammatory response [101]. Blackberry leaf pectin exerted a significant anti-inflammatory potential, as it decreased the induced levels of pro-inflammatory cytokines (TNF-α and IL-6) in a dose-dependent manner [44]. Furthermore, pectin from blackberry leaves inhibited NO secretion by suppressing the expression of the iNOS gene [44]. Lotus leaf pectins significantly stimulated macrophage cell-line proliferation, increased macrophage activation (stimulated NO release and stimulated TNF-α, IL-1β, and IL-6 secretion in a dose-dependent manner), and triggered and increased phagocytic activity [90]. Compounds of natural origin, such as pectins, which enhance the regulation of the immune system and the differentiation of immune cells, become helpful in the treatment of immunological changes and even diseases related to them.

#### 3.4.3. Antitumor Activity of Pectins

The cytotoxic effect toward cancer cells is one of the most valuable potentials for the clinical application of pectins since classic chemotherapeutics are toxic to normal cells as well. The optimal antitumor effect of pectins is probably the result of a specific structural characterization, i.e., (i) the conformation adopted by the galactan and arabinan side chains in cooperation with the HG/RG-I backbone is required, and it maximizes the availability of neutral side chains for cellular receptors to induce cell detachment [55,84]; (ii) →4)-β-D-Galp-(1→ units on the branched chain in the RG-I region inhibits the function of galactin-3 [43,55,128]; (iii) relatively low Mw and DE may increase activity [84]. The immunostimulatory activity of pectins (via initiation of macrophages in the immune response) is considered to be the main factor in the antitumor effect [87]. Furthermore, the antioxidant potential of pectins is positively associated with antitumor efficacy because pectins scavenge and/or reduce the activity of reactive oxygen species in cancer cells and directly reduce growth [65]. The opposite therapeutic strategy for pectins towards cancer cells is to facilitate an increase of ROS to a toxic level beyond the breaking point that forces cancer cells to activate ROS-induced cell-death pathways [43]. In this sense, the cytotoxic effect of the HMP of the gabiroba fruit pulp was expressed by a decrease in the number of adherent human glioblastoma cell lines (U251-MG), induced significant changes in cell morphology, and caused increased levels of cellular ROS that alter the cellular redox status [43]. Amaral et al. [43] linked this effect to the large fraction of RG-I, a high extension of neutral side chains, and exceptionally large amounts of arabinose (54.5%) and galactose (7.6%). The specific HMP structure of papaya fruit pulp, which has relatively low Mw, and the structure of the arabinogalactan type II (AGII) branches determined its antiproliferative activity in cancer cells of three types of cancer cell lines (colon cancer cells (HCT116 and HT29) and the prostate cancer cell line (PC3)) [55]. Papaya pectin disrupted the interaction between cancer cells and extracellular matrix proteins, increased cell separation, disorganized cell cycle, and promoted apoptosis/necroptosis [55]. HMPs from mandarin fruit peel and apple pomace significantly inhibited HT29 cell lines in a dose-dependent manner [35].

The LMP of the fig peel presented immunostimulatory-induced antiproliferative activity in the growth of lung cancer cells (A549) and hepatocellular carcinoma cells (HepG2) in vitro, which correlated with antioxidant activity [65]. The LMP of sugar beet root induced apoptosis of colon cancer cells (HT19 and DLD1) [84]. The LMP of the grapefruit peel inhibited the proliferation of human neuroblastoma cells (SH-SY5Y) [59]. Blackberry leaf pectin reduced the proliferation of breast cancer cells (MCF-7) and liver cancer cells (Bel-7402) in a dose- and time-dependent manner [44]. All the pectins discussed were selective and did not show cytotoxicity for healthy human cells. In this context, the by-products of waste pectins could be effective antitumor candidates and may exert a synergistic effect in combination with conventional anticancer agents.

#### 3.4.4. Antimicrobial Activity of Pectins

The antimicrobial activity of pectins is useful in the treatment of pathogen-induced bacteremia, skin, and soft tissue infections, and even infections related to prosthetic implants that threaten human health. Pectins could also be used as an antibiofilm agent in chronic decubitus ulcers, endotracheal tubes in intubated patients, urinary catheters, and surgical sites [9]. Moreover, pectins could be an interesting antimicrobial solution oriented for an oral application that is convenient for patients. The LMP–flavonoid complex of grapefruit albedo showed very high antibacterial activity in vitro against *Staphylococcus aureus* and *Pseudomonas aeruginosa* strains at a concentration as low as 15 mg/mL [9]. This is an important finding because infections caused by the antibiotic-resistant opportunistic strain of *S. aureus* or *P. aeruginosa* are very difficult to treat. These strains are extremely resistant to innate immune antimicrobial peptides and several antibiotics, often causing high mortality in critically ill and immunosuppressed patients [9]. Presentato et al. [9] suggested that this exceptional antibacterial activity of grapefruit pectin is the result of synergy between pectin and flavonoids (nobiletin and tangeretin), derived from plant material along with pectin. In fact, these polyphenols are capable of inhibiting key enzyme activities (GTPase) and altering the filaments of temperature-sensitive mutant Z protein synthesis in bacteria, eventually leading to cell death [129]. Furthermore, Presentanto [9] proposed that the mechanism of pectin antimicrobial activity relies on the induction of defects during the cell division process and the alteration of the morphology of bacterial cells resulting in elongated or collapsed cells. In the case of this particular grapefruit phenol–pectin complex, this vesicle’s process can even be enhanced by aromatic compounds that penetrate nonpolar regions of the cell membrane of bacteria, resulting in physiological fluid changes [130]. Amorim et al. [101] suggested that LMP from cocoa pods exerts indirect microbicidal activity. Cocoa pectin activates the nitric oxide pathway in murine peritoneal macrophages in vitro, and the NO produced is highly toxic to microorganisms [101]. Jridi et al. [32] confirmed that the HMP of the orange fruit peel incorporated in composite films increases antibacterial activity toward pathogenic *Micrococcus luteus*, *Bacillus cereus*, *Enterococcus faecalis,* and *Enterobacter* sp. in vitro. The orange pectin composite used as a wrapping film for Ricotta cheese delayed and restricted the growth of yeast and mold on its surface [32]. Apparently, orange pectin helped form a barrier against bacterial proliferation and oxygen diffusion [32].

#### 3.4.5. Pectins as Prebiotics

Pectins are non-digestible food ingredients susceptible to hydrolysis by glycosidases to monomers that might later be fermented to short-chain fatty acids (acetic, lactic butyric, and propionic acids) and different gasses by microorganisms [131]. If these substances selectively promote the growth and well-being of the host probiotic microorganisms in the intestine, mainly Bifidobacterium and Lactobacillus spp., they are considered prebiotics. However, it should be noted that pectin could increase the viscosity of the gastrointestinal contents and diminish the utilization of nutrients by the microflora. Thus, it is important to verify their prebiotic potential [3]. The HMP of the lemon fruit peel and the LMP of the sugar beet root positively modulated the fermentation dynamics of the gastrointestinal microbiota in vitro in fermentation assays with human fecal inocula [30]. Both pectins increased populations of bifidobacteria and lactobacilli species and allowed for the growth of clostridia and Bacteroides [30]. Stronger bifidogenic effects were observed for sugar beet pectin, while a higher increase in the number of lactobacilli was observed in cultures with lemon peel pectin [30]. LMPs extracted from wastewater discharged from the canning process of satsuma mandarin positively alleviated gut microbiota dysbiosis in obesity-induced mice [74]. Satsuma mandarin pectin promoted the growth of Bacteroidia species (*Bacteroides caecimuris*, *Bacteroides ovatus*), *Barnesiella*, *Butyrivibrio*, *Roseburia*, *Prophyromonadaceae*, *Flavonifractor*, *Acetivibrio*, *Clostridium* cluster IV, and the Ruminococcaceae family (Ruminococcus spp., Butyricicoccus spp.) [73,74]. Mao et al. suggested a strong effect of the structure (Mw, branching) and composition (RG-I/HG/arabinose content) of pectin on the modulation of the gut microbiota. Although intestinal bacteria are capable of degrading most of the main plant glycans, including the most complex structures of polysaccharides abundant in RG-I (preferably) and RG-II regions, pectin with a low Mw, high content of neutral sugars, and fewer side chains are more effective in promoting the growth of probiotics [74]. This is probably a result of the higher solubility and accessibility of the pectic backbone to microbial degrading enzymes. Moreover, it was proven that bacteria of the Ruminococcaceae family, especially Ruminococcus spp. and Butyricicoccus spp., degrade the arabinan side chain the most effectively. The LMP of the banana fruit peel was confirmed as a prebiotic as it increased the initial microbial load of natural yogurt [63]. The LMP from buttonwood leaves also promoted the growth of *L. paracasei* and *L. rhamnosus* in vitro [97]. The well-being of the gastrointestinal tract microbiota also depends on effective defecation. Pectin is considered food fiber that prevents constipation and helps with a gastrointestinal disorder characterized by a hard texture and difficult excretion of the stool [132]. The edible burdock root derived from HMP alleviates constipation in vivo in the diphenoxylate-induced constipation model in mice [12]. Edible burdock pectin restored small intestinal movement and significantly increased feces weight in vivo in mice, which had no effect on body weight and was non-toxic [12]. Li et al. [12] attributed the laxative effect of edible burdock pectin to a high WHC as it increases the fecal water content that allows for the evacuation of the feces. Pectins can promote the maintenance of healthy gut homeostasis and indirectly contribute to the reduction of the risks of colon cancer and ulcerative colitis. Therefore, they are considered prebiotic ingredients for a variety of functional foods.

#### 3.4.6. Pectins as Metabolic Modulators

Pectins are capable of directly or indirectly modulating malfunctions of glucose, insulin, cholesterol, or short-chain fatty acid (SCFA) metabolism. Pectins in the gastrointestinal system form a complex three-dimensional network whose structure depends on the chemical and physical properties of these biopolymers, mainly DE, Mw, GalA, and glucose content [133]. Pectins increase the viscosity of ingested food, which often hinders diffusion and access of digestive enzymes, but pectins themselves are resistant to most hydrolytic enzymes. Digestive enzymes, i.e., α-amylase and α-glucosidase, are responsible for the degradation of dietary carbohydrates into simple monosaccharides, and as such they are one of the first targets for pectins to delay glucose absorption and decrease glycemic levels [134]. The HMP of the red chilto fruit peels was characterized by hypoglycemic activity in vivo in *Sacharomyces cerevisae* species [78]. Chilto pectin inhibited the activity of α-amylase and retarded glucose diffusion through glucose transporters in yeast cells in a dose response [78]. The HMP of the orange peel showed an anti-diabetic effect in vivo in type 2 diabetic rats [34]. Orange pectin was effective in the regulation of glucose metabolism in a dose-dependent manner: decreased fasting blood glucose levels, improved glucose tolerance, and significantly reduced insulin resistance [34]. Liu et al. [34] suggested that orange pectin regulated glucose metabolism through the PI3K/Akt-signaling pathway and could be an effective alternative to metformin, a first-choice drug against type 2 diabetes mellitus.

Moreover, pectins indirectly contribute to the control level of cholesterol by binding covalently to the active site of pancreatic lipase, forming a stable complex that reduces the hydrolysis of triglycerides, so less SCFA and glycerol are produced and, consequently, cholesterol levels decrease [135]. In this regard, pectins of high Mw have a greater effect on lipase inactivation capacity than pectins of low Mw [48]. The HMP of grapefruit peels of the relatively high Mw (~280 kDa) significantly inhibited the activity of lipase in vitro [56]. The HMP of the orange peel indirectly reduced hyperlipidemia and improved liver glycogen synthesis (decreased triglycerides, total cholesterol, low-density cholesterol (LDL), and increased high-density cholesterol (HDL)) in vivo in type 2 diabetic rats [34]. On the other hand, a direct cholesterol-lowering potential was observed for HMP from the dragon fruit peel [48]. Cholesterol adsorbed on a heterogeneous surface of dragon fruit pectin through van der Waals interactions as a result of hydrophobic affinity between the lipophilic compound and esterified GalA moieties in the pectin chain [48]. Zaid et al. [48] suggested that interactions between pectin and cholesterol followed the solid–liquid sorption mechanism and were positively correlated with the concentration of pectin. Such a physisorption of cholesterol in the digestive tract reduces serum cholesterol.

Furthermore, pectins have been found to be useful in maintaining proper body weight and preventing obesity, which is often associated with altered intestinal bacterial diversity compared to healthy intestinal microbiota [136]. Gut bacteria ferment neutral sugar-branching chains of RG-I regions (arabinan, galactan, and arabinogalactan) to short-chain fatty acids (SCFA) [137]. Specifically, GalA and xylose fermentation promote the production of acetate and butyrate, while arabinose and glucose fermentation promote the production of propionate [138]. The anti-obesity effect of SCFA is associated with the induction of intestinal hormone release, the enhancement of intestinal barrier function, the inhibition of fat accumulation and adipocyte dysfunction, activating PGC-1α enzyme (main regulator of mitochondrial biogenesis) for fat browning, and the regulation of energy homeostasis during fermentation [139]. In this regard, a dominant agent is butyrate, which modulates healthy microbiota to reduce the risk of obesity. Zhu et al. [73] introduced LMPs from wastewater (HCl_aq_) discharged from the satsuma mandarin canning process as a new, multifunctional anti-obesity agent. Satsuma mandarin pectin indirectly alleviated obesity in vivo in mice fed high-fat diets through the regulation of intestinal dysbiosis; that is, increased abundance of *Butyrivibrio*, *Roseburia*, *Barnesiella*, *Flavonifractor*, *Acetivibrio,* and *Clostridium* cluster IV [73]. Balancing gut microorganisms’ well-being resulted in (i) improved insulin tolerance, (ii) reduced macrosteatosis and ballooning of hepatocytes in the liver, (iii) alleviated hyperglycemia and hyperlipidemia, (iv) suppressed adipocyte hypertrophy, and (v) promoted browning of white adipose tissues [97]. These individual effects combined, in general, to prevent obesity. Pectins are profound candidate modulators to treat metabolic disorders and, thus, are considered valuable and safe additives for food and beverages, reducing the need for medication.

#### 3.4.7. Pectins as Protective Agents

The LMP–flavonoid complex of grapefruit albedo demonstrated a cardioprotective effect ex vivo in the hearts of rats damaged by myocardial ischemia/perfusion episodes [57]. The grapefruit pectin–naringenin complex recovered hearts to pre-ischemic parameters [57]. Specifically, naringenin in the grapefruit complex depolarized isolated cardiac mitochondria and decreased calcium uptake into the mitochondrial matrix in a dose-dependent manner [57]. This suggests that anti-ischemic cardioprotective activity is based on the stimulation of potassium channels located on the sarcolemmal and inner membranes of cardiac mitochondria. At the same time, the carbohydrate component provided exceptional solubility in aqueous conditions of the grapefruit pectin complex due to its specific structure; that is, low DE and a high content of RG-I regions rich in galactose and arabinose units. Flori et al. [57] emphasized that the activity of the grapefruit pectin complex is comparable to the activity of naringin/naringenin in terms of cardiovascular beneficial effects [140], but at a dose several times lower due to poor bioavailability of flavonoid at a systemic level [57].

HMPs from pumpkin fruit pulp protected cell lines HT-29 and MDCK1 (used as models of the intestinal epithelium) from toxicity induced by Cd and Hg. The cytoprotective effect of pumpkin pectin was greater against Cd ions [87]. Torkova et al. [87] suggest that the cytoprotective effect of pumpkin pectin is the result of the overall structure of the polysaccharide as they did not observe a correlation between the cytoprotective effects and the DE, Mw, or GalA content [87]. Recent studies on HMP of the red chilto fruit peel evaluated toxicity in vivo on the model organisms Artemia salina, a marine zooplankton crustacean, and a nematode *Caenoharbitis elegant* [78]. In acute toxicity assays, the viability of both organisms remained unaffected, even under 5% red chilto pectin concentration [78]. Therefore, Orqueda et al. [78] suggested that red chilto pectin could be classified as nontoxic according to the WHO classification for hazardous substances [132].

LMP polyphenol complexes from wild strawberry leaves inhibit the intrinsic pathway of the coagulation cascade in vitro but do not affect the extrinsic coagulation pathway [39,40]. Studies on the mechanism of anticoagulant activity of wild strawberry polyphenolic glycoconjugates revealed that they are indirect factor Xa inhibitors mediated by antithrombin [39,40]. Pawlaczyk et al. [39,40] did not observe any correlation between the antithrombotic effect and the content of Mw and GalA in the studied pectic complexes. These findings suggest that these polyphenol glycoconjugates can be effective anticoagulants that are not associated with some side effects such as uncontrolled bleeding [39,40].

## 4. Conclusions

Industrial waste from fruits, vegetables, and herbs and nuts consists of both edible (pulp, pomace, etc.) and inedible (peel, leaves, roots, shells, pods, etc.) by-products generated during large-scale processes. However, the total waste from the food processing industry consists of up to 90% by-products of fruit processing, of which almost 50% is fruit peel. Pectins can be easily recovered from this biomass since polysaccharides are the most abundant components of plant cell walls. Pectins, sometimes referred to as “soluble dietary fiber”, unite a group of substances that do not have a strictly defined structure. These biopolymers differ structurally to a large extent in terms of Mw, DE, HG/RG-I ratio, and even monosaccharide composition, including GalA content. In reality, this strongly depends on the plant source and its stage of maturity, as well as on the separation technique. Because the chemical diversity is quantitatively reflected in the characteristics of these biopolymers, the elucidation of the structural properties of pectins that are actually involved in their functional properties and biological activity is highly desirable. Furthermore, the constant progress in pectin-separation techniques is eliminating the environmentally demanding conventional extraction techniques and transforming them into environmentally beneficial methods such as room-temperature liquid–liquid separation assisted by ultrasound, microwaves, high pressure, enzymes, etc. Green solvents, such as supercritical fluids and deep eutectic solvents, consequently reduce the excessive use of conventional aggressive and/or organic solvents to maximize the yield and quality of pectin recovery and minimize the negative impact on the environment.

Pectins are known as emerging compounds, especially for food and pharmacological applications due to their easy availability, biocompatibility, biodegradability and valuable rheological, interfacial, functional, and biological properties. This review provides an in-depth insight into the recent developments in multifunctional pectins derived from food wastes and by-products described in the last 10 years and refers to typical (such as citrus fruits, apples, or sugar beets) and alternative (such as chocolate wine fruits, palm fruits, melon and watermelon, banana, mango, jackfruit, passion fruit, edible burdock, broccoli, carrot, tomato, eggplant, corn, husks, or peels) biomass. These remarks help to systematise the dispute between the type of food waste and the structure of pectin with its functionality and, finally, its pharmaceutical and technological applicability as formulation texturizers, biologically active agents, and even new advanced biomaterials.

## References

[1] Banerjee J., Singh R., Vijayaraghavan R., MacFarlane D., Patti A.F., Arora A. (2017). Bioactives from Fruit Processing Wastes: Green Approaches to Valuable Chemicals. Food Chem..

[2] Zema D.A., Calabrò P.S., Folino A., Tamburino V., Zappia G., Zimbone S.M. (2018). Valorisation of Citrus Processing Waste: A Review. Waste Manag..

[3] Naqash F., Masoodi F.A., Rather S.A., Wani S.M., Gani A. (2017). Emerging Concepts in the Nutraceutical and Functional Properties of Pectin—A Review. Carbohydr. Polym..

[4] FAO (2022). World Food and Agriculture—Statistical Yearbook 2022.

[5] Jha A., Kumar A. (2019). Biobased Technologies for the Efficient Extraction of Biopolymers from Waste Biomass. Bioprocess. Biosyst. Eng..

[6] Banožić M., Babić J., Jokić S. (2020). Recent Advances in Extraction of Bioactive Compounds from Tobacco Industrial Waste-a Review. Ind. Crops Prod..

[7] Sharma P., Vishvakarma R., Gautam K., Vimal A., Kumar Gaur V., Farooqui A., Varjani S., Younis K. (2022). Valorization of Citrus Peel Waste for the Sustainable Production of Value-Added Products. Bioresour. Technol..

[8] Santra S., Das M., Karmakar S., Banerjee R. (2023). NADES Assisted Integrated Biorefinery Concept for Pectin Recovery from Kinnow (*Citrus Reticulate*) Peel and Strategic Conversion of Residual Biomass to L(+) Lactic Acid. Int. J. Biol. Macromol..

[9] Presentato A., Piacenza E., Scurria A., Albanese L., Zabini F., Meneguzzo F., Nuzzo D., Pagliaro M., Martino D.C., Alduina R. (2020). A New Water-Soluble Bactericidal Agent for the Treatment of Infections Caused by Gram-Positive and Gram-Negative Bacterial Strains. Antibiotics.

[10] Jin M.Y., Li M.Y., Huang R.M., Wu X.Y., Sun Y.M., Xu Z.L. (2021). Structural Features and Anti-Inflammatory Properties of Pectic Polysaccharides: A Review. Trends Food Sci. Technol..

[11] Humerez-Flores J.N., Verkempinck S.H.E., Kyomugasho C., Moldenaers P., Van Loey A.M., Hendrickx M.E. (2021). Modified Rhamnogalacturonan-Rich Apple Pectin-Derived Structures: The Relation between Their Structural Characteristics and Emulsifying and Emulsion-Stabilizing Properties. Foods.

[12] Li K., Zhu L., Li H., Zhu Y., Pan C., Gao X., Liu W. (2019). Structural Characterization and Rheological Properties of a Pectin with Anti-Constipation Activity from the Roots of *Arctium Lappa* L. Carbohydr. Polym..

[13] Abodinar A., Smith A.M., Morris G.A. (2014). A Novel Method to Estimate the Stiffness of Carbohydrate Polyelectrolyte Polymers Based on the Ionic Strength Dependence of Zeta Potential. Carbohydr. Polym..

[14] Liang W.-L., Liao J.-S., Qi J.-R., Jiang W.-X., Yang X.-Q. (2022). Physicochemical Characteristics and Functional Properties of High Methoxyl Pectin with Different Degree of Esterification. Food Chem..

[15] Gavahian M., Mathad G.N., Pandiselvam R., Lin J., Sun D.W. (2021). Emerging Technologies to Obtain Pectin from Food Processing By-Products: A Strategy for Enhancing Resource Efficiency. Trends Food Sci. Technol..

[16] Marić M., Grassino A.N., Zhu Z., Barba F.J., Brnčić M., Rimac Brnčić S. (2018). An Overview of the Traditional and Innovative Approaches for Pectin Extraction from Plant Food Wastes and By-Products: Ultrasound-, Microwaves-, and Enzyme-Assisted Extraction. Trends Food Sci. Technol..

[17] Wicker L., Kim Y., Kim M.J., Thirkield B., Lin Z., Jung J. (2014). Pectin as a Bioactive Polysaccharide—Extracting Tailored Function from Less. Food Hydrocoll..

[18] Singhal S., Swami Hulle N.R. (2022). Citrus Pectins: Structural Properties, Extraction Methods, Modifications and Applications in Food Systems—A Review. Appl. Food Res..

[19] Georgiev Y., Ognyanov M., Yanakieva I., Kussovski V., Kratchanova M. (2012). Isolation, Characterization and Modification of Citrus Pectins. J. BioSci. Biotech..

[20] Kumar S., Konwar J., Purkayastha M.D., Kalita S., Mukherjee A., Dutta J. (2023). Current Progress in Valorization of Food Processing Waste and By-Products for Pectin Extraction. Int. J. Biol. Macromol..

[21] Rahmani Z., Khodaiyan F., Kazemi M., Sharifan A. (2020). Optimization of Microwave-Assisted Extraction and Structural Characterization of Pectin from Sweet Lemon Peel. Int. J. Biol. Macromol..

[22] Yuliarti O., Goh K.K.T., Matia-Merino L., Mawson J., Brennan C. (2015). Extraction and Characterisation of Pomace Pectin from Gold Kiwifruit (*Actinidia Chinensis*). Food Chem..

[23] Petkowicz C.L.O., Williams P.A. (2020). Pectins from Food Waste: Characterization and Functional Properties of a Pectin Extracted from Broccoli Stalk. Food Hydrocoll..

[24] Begum R., Aziz M.G., Yusof Y.A., Saifullah M., Uddin M.B. (2021). Evaluation of Gelation Properties of Jackfruit (*Artocarpus Heterophyllus*) Waste Pectin. Carbohydr. Polym. Technol. Appl..

[25] Li W.J., Fan Z.G., Wu Y.Y., Jiang Z.G., Shi R.C. (2019). Eco-Friendly Extraction and Physicochemical Properties of Pectin from Jackfruit Peel Waste with Subcritical Water. J. Sci. Food Agric..

[26] Petkowicz C.L.O., Vriesmann L.C., Williams P.A. (2017). Pectins from Food Waste: Extraction, Characterization and Properties of Watermelon Rind Pectin. Food Hydrocoll..

[27] Méndez D.A., Schroeter B., Martínez-Abad A., Fabra M.J., Gurikov P., López-Rubio A. (2023). Pectin-Based Aerogel Particles for Drug Delivery: Effect of Pectin Composition on Aerogel Structure and Release Properties. Carbohydr. Polym..

[28] Liu Z., Wu S., Zuo H., Lin J., Zheng H., Lei H., Yu Q., Wu X., Guo Z. (2023). Freeze-Drying Pretreatment of Watermelon Peel to Improve the Efficiency of Pectin Extraction: RSM Optimization, Extraction Mechanism, and Characterization. Int. J. Biol. Macromol..

[29] Dominiak M., Søndergaard K.M., Wichmann J., Vidal-Melgosa S., Willats W.G.T., Meyer A.S., Mikkelsen J.D. (2014). Application of Enzymes for Efficient Extraction, Modification, and Development of Functional Properties of Lime Pectin. Food Hydrocoll..

[30] Gómez B., Gullón B., Yáñez R., Schols H., Alonso J.L. (2016). Prebiotic Potential of Pectins and Pectic Oligosaccharides Derived from Lemon Peel Wastes and Sugar Beet Pulp: A Comparative Evaluation. J. Funct. Foods.

[31] Nuzzo D., Cristaldi L., Sciortino M., Albanese L., Scurria A., Zabini F., Lino C., Pagliaro M., Meneguzzo F., Di Carlo M. (2020). Exceptional Antioxidant, Non-Cytotoxic Activity of Integral Lemon Pectin from Hydrodynamic Cavitation. ChemistrySelect.

[32] Jridi M., Abdelhedi O., Salem A., Kechaou H., Nasri M., Menchari Y. (2020). Physicochemical, Antioxidant and Antibacterial Properties of Fish Gelatin-Based Edible Films Enriched with Orange Peel Pectin: Wrapping Application. Food Hydrocoll..

[33] Zhao W., Xu Y., Dorado C., Chau H.K., Hotchkiss A.T., Cameron R.G. (2024). Modification of Pectin with High-Pressure Processing Treatment of Fresh Orange Peel before Pectin Extraction: Part I. The Effects on Pectin Extraction and Structural Properties. Food Hydrocoll..

[34] Liu Y., Dong M., Yang Z., Pan S. (2016). Anti-Diabetic Effect of Citrus Pectin in Diabetic Rats and Potential Mechanism via PI3K/Akt Signaling Pathway. Int. J. Biol. Macromol..

[35] Wang X., Chen Q., Lü X. (2014). Pectin Extracted from Apple Pomace and Citrus Peel by Subcritical Water. Food Hydrocoll..

[36] Mahmoud M.H., Abu-Salem F.M., Azab D.E.S.H. (2022). A Comparative Study of Pectin Green Extraction Methods from Apple Waste: Characterization and Functional Properties. Int. J. Food Sci..

[37] Gawkowska D., Cybulska J., Zdunek A. (2018). Cross-Linking of Sodium Carbonate-Soluble Pectins from Apple by Zinc Ions. Carbohydr. Polym..

[38] Gurev A., Cesko T., Dragancea V., Ghendov-Mosanu A., Pintea A., Sturza R. (2023). Ultrasound-and Microwave-Assisted Extraction of Pectin from Apple Pomace and Its Effect on the Quality of Fruit Bars. Foods.

[39] Pawlaczyk-Graja I., Balicki S., Wilk K.A. (2019). Effect of Various Extraction Methods on the Structure of Polyphenolic-Polysaccharide Conjugates from *Fragaria Vesca* L. Leaf. Int. J. Biol. Macromol..

[40] Pawlaczyk-Graja I., Balicki S., Ziewiecki R., Capek P., Matulová M., Wilk K.A. (2020). New Isolation Process for Bioactive Food Fiber from Wild Strawberry Leaf. Biochem. Eng. J..

[41] Orqueda M.E., Méndez D.A., Martínez-Abad A., Zampini C., Torres S., Isla M.I., López-Rubio A., Fabra M.J. (2022). Feasibility of Active Biobased Films Produced Using Red Chilto Wastes to Improve the Protection of Fresh Salmon Fillets via a Circular Economy Approach. Food Hydrocoll..

[42] Assoi S., Konan K., Walker L.T., Holser R., Agbo G.N., Dodo H., Wicker L. (2014). Functionality and Yield of Pectin Extracted from Palmyra Palm (*Borassus Aethiopum* Mart) Fruit. LWT.

[43] da Costa Amaral S., Barbieri S.F., Ruthes A.C., Bark J.M., Brochado Winnischofer S.M., Silveira J.L.M. (2019). Cytotoxic Effect of Crude and Purified Pectins from *Campomanesia xanthocarpa* Berg on Human Glioblastoma Cells. Carbohydr. Polym..

[44] Zhang T.T., Lu C.L., Jiang J.G., Wang M., Wang D.M., Zhu W. (2015). Bioactivities and Extraction Optimization of Crude Polysaccharides from the Fruits and Leaves of *Rubus chingii* Hu. Carbohydr. Polym..

[45] Espinal-Ruiz M., Restrepo-Sánchez L.P., Narváez-Cuenca C.E., McClements D.J. (2016). Impact of Pectin Properties on Lipid Digestion under Simulated Gastrointestinal Conditions: Comparison of Citrus and Banana Passion Fruit (*Passiflora tripartita* Var. *mollissima*) Pectins. Food Hydrocoll..

[46] El Fihry N., El Mabrouk K., Eeckhout M., Schols H.A., Filali-Zegzouti Y., Hajjaj H. (2022). Physicochemical and Functional Characterization of Pectin Extracted from Moroccan Citrus Peels. LWT.

[47] Çavdaroğlu E., Büyüktaş D., Farris S., Yemenicioğlu A. (2023). Novel Edible Films of Pectins Extracted from Low-Grade Fruits and Stalk Wastes of Sun-Dried Figs: Effects of Pectin Composition and Molecular Properties on Film Characteristics. Food Hydrocoll..

[48] Zaid R.M., Mishra P., Wahid Z.A., Sakinah A.M.M. (2019). Hylocereus Polyrhizus Peel’s High-Methoxyl Pectin: A Potential Source of Hypolipidemic Agent. Int. J. Biol. Macromol..

[49] Ghoshal G., Negi P. (2020). Isolation of Pectin from Kinnow Peels and Its Characterization. Food Bioprod. Process..

[50] Wathoni N., Yuan Shan C., Yi Shan W., Rostinawati T., Indradi R.B., Pratiwi R., Muchtaridi M. (2019). Characterization and Antioxidant Activity of Pectin from Indonesian Mangosteen (*Garcinia mangostana* L.) Rind. Heliyon.

[51] Raji Z., Khodaiyan F., Rezaei K., Kiani H., Hosseini S.S. (2017). Extraction Optimization and Physicochemical Properties of Pectin from Melon Peel. Int. J. Biol. Macromol..

[52] Yu N., Wang X., Ning F., Jiang C., Li Y., Peng H., Xiong H. (2019). Development of Antibacterial Pectin from *Akebia trifoliata* Var. *Australis* Waste for Accelerated Wound Healing. Carbohydr. Polym..

[53] Lira-Ortiz A.L., Reséndiz-Vega F., Ríos-Leal E., Contreras-Esquivel J.C., Chavarría-Hernández N., Vargas-Torres A., Rodríguez-Hernández A.I. (2014). Pectins from Waste of Prickly Pear Fruits (*Opuntia albicarpa* Scheinvar ‘Reyna’): Chemical and Rheological Properties. Food Hydrocoll..

[54] Xie F., Zhang W., Lan X., Gong S., Wu J., Wang Z. (2018). Effects of High Hydrostatic Pressure and High Pressure Homogenization Processing on Characteristics of Potato Peel Waste Pectin. Carbohydr. Polym..

[55] Prado S.B.R.D., Ferreira G.F., Harazono Y., Shiga T.M., Raz A., Carpita N.C., Fabi J.P. (2017). Ripening-Induced Chemical Modifications of Papaya Pectin Inhibit Cancer Cell Proliferation. Sci. Rep..

[56] Wang W., Ma X., Jiang P., Hu L., Zhi Z., Chen J., Ding T., Ye X., Liu D. (2016). Characterization of Pectin from Grapefruit Peel: A Comparison of Ultrasound-Assisted and Conventional Heating Extractions. Food Hydrocoll..

[57] Flori L., Albanese L., Calderone V., Meneguzzo F., Pagliaro M., Ciriminna R., Zabini F., Testai L. (2022). Cardioprotective Effects of Grapefruit IntegroPectin Extracted via Hydrodynamic Cavitation from By-Products of *Citrus* Fruits Industry: Role of Mitochondrial Potassium Channels. Foods.

[58] Scordino M., Urone G., Frinchi M., Valenza C., Bonura A., Cipollina C., Ciriminna R., Meneguzzo F., Pagliaro M., Mudò G. (2024). Anti-Apoptotic and Anti-Inflammatory Properties of Grapefruit IntegroPectin on Human Microglial HMC3 Cell Line. Cells.

[59] Nuzzo D., Scordino M., Scurria A., Giardina C., Giordano F., Meneguzzo F., Mudò G., Pagliaro M., Picone P., Attanzio A. (2021). Protective, Antioxidant and Antiproliferative Activity of Grapefruit Integropectin on Sh-Sy5y Cells. Int. J. Mol. Sci..

[60] Golbargi F., Gharibzahedi S.M.T., Zoghi A., Mohammadi M., Hashemifesharaki R. (2021). Microwave-Assisted Extraction of Arabinan-Rich Pectic Polysaccharides from Melon Peels: Optimization, Purification, Bioactivity, and Techno-Functionality. Carbohydr. Polym..

[61] Rivadeneira J.P., Castillo-Israel K.A.T., Wu T. (2023). Physicochemical Characteristics, Rheology, and Emulsifying Properties of Ultrasound-Extracted Pectin from “saba” Banana Peel. Food Res..

[62] Wang M., Huang B., Fan C., Zhao K., Hu H., Xu X., Pan S., Liu F. (2016). Characterization and Functional Properties of Mango Peel Pectin Extracted by Ultrasound Assisted Citric Acid. Int. J. Biol. Macromol..

[63] Rivadeneira J.P., Wu T., Ybanez Q., Dorado A.A., Migo V.P., Nayve F.R.P., Castillo-Israel K.A.T. (2020). Microwave-Assisted Extraction of Pectin from “Saba” Banana Peel Waste: Optimization, Characterization, and Rheology Study. Int. J. Food Sci..

[64] Rodsamran P., Sothornvit R. (2019). Preparation and Characterization of Pectin Fraction from Pineapple Peel as a Natural Plasticizer and Material for Biopolymer Film. Food Bioprod. Process..

[65] Gharibzahedi S.M.T., Smith B., Guo Y. (2019). Ultrasound-Microwave Assisted Extraction of Pectin from Fig (*Ficus carica* L.) Skin: Optimization, Characterization and Bioactivity. Carbohydr. Polym..

[66] Zhao L., Wu L., Li L., Zhu J., Chen X., Zhang S., Li L., Yan J.K. (2023). Physicochemical, Structural, and Rheological Characteristics of Pectic Polysaccharides from Fresh Passion Fruit (*Passiflora edulis* f. *Flavicarpa* L.) Peel. Food Hydrocoll..

[67] Lee T., Chang Y.H. (2020). Structural, Physicochemical, and in-Vitro Release Properties of Hydrogel Beads Produced by Oligochitosan and de-Esterified Pectin from Yuzu (*Citrus junos*) Peel as a Quercetin Delivery System for Colon Target. Food Hydrocoll..

[68] Zhang H., Chen J., Li J., Wei C., Ye X., Shi J., Chen S. (2018). Pectin from Citrus Canning Wastewater as Potential Fat Replacer in Ice Cream. Molecules.

[69] Guo X., Han D., Xi H., Rao L., Liao X., Hu X., Wu J. (2012). Extraction of Pectin from Navel Orange Peel Assisted by Ultra-High Pressure, Microwave or Traditional Heating: A Comparison. Carbohydr. Polym..

[70] Presentato A., Scurria A., Albanese L., Lino C., Sciortino M., Pagliaro M., Zabini F., Meneguzzo F., Alduina R., Nuzzo D. (2020). Superior Antibacterial Activity of Integral Lemon Pectin Extracted via Hydrodynamic Cavitation. ChemistryOpen.

[71] Nuzzo D., Picone P., Giardina C., Scordino M., Mudò G., Pagliaro M., Scurria A., Meneguzzo F., Ilharco L.M., Fidalgo A. (2021). New Neuroprotective Effect of Lemon Integropectin on Neuronal Cellular Model. Antioxidants.

[72] Méndez D.A., Martínez-Abad A., Martínez-Sanz M., López-Rubio A., Fabra M.J. (2023). Tailoring Structural, Rheological and Gelling Properties of Watermelon Rind Pectin by Enzymatic Treatments. Food Hydrocoll..

[73] Zhu K., Mao G., Wu D., Yu C., Cheng H., Xiao H., Ye X., Linhardt R.J., Orfila C., Chen S. (2020). Highly Branched RG-I Domain Enrichment Is Indispensable for Pectin Mitigating against High-Fat Diet-Induced Obesity. J. Agric. Food Chem..

[74] Mao G., Li S., Orfila C., Shen X., Zhou S., Linhardt R.J., Ye X., Chen S. (2019). Depolymerized RG-I-Enriched Pectin from Citrus Segment Membranes Modulates Gut Microbiota, Increases SCFA Production, and Promotes the Growth of *Bifidobacterium* Spp., *Lactobacillus* Spp. and *Faecalibaculum* Spp. Food Funct..

[75] Muhammad K., Nur N.I., Gannasin S.P., Adzahan N.M., Bakar J. (2014). High Methoxyl Pectin from Dragon Fruit (Hylocereus Polyrhizus) Peel. Food Hydrocoll..

[76] Gawkowska D., Ciesla J., Zdunek A., Cybulska J. (2019). The Effect of Concentration on the Cross-Linking and Gelling of Sodium Carbonate-Soluble Apple Pectins. Molecules.

[77] Mada T., Duraisamy R., Abera A., Guesh F. (2022). Effect of Mixed Banana and Papaya Peel Pectin on Chemical Compositions and Storage Stability of Ethiopian Traditional Yoghurt (Ergo). Int. Dairy. J..

[78] Orqueda M.E., Zampini I.C., Torres S., Isla M.I. (2023). Functional Characterization and Toxicity of Pectin from Red Chilto Fruit Waste (Peels). Plants.

[79] Yang J.S., Mu T.H., Ma M.M. (2018). Extraction, Structure, and Emulsifying Properties of Pectin from Potato Pulp. Food Chem..

[80] Idrovo Encalada A.M., Pérez C.D., Gerschenson L.N., Rojas A.M., Fissore E.N. (2019). Gelling Pectins from Carrot Leftovers Extracted by Industrial-Enzymes with Ultrasound Pretreatment. LWT.

[81] Alancay M.M., Lobo M.O., Quinzio C.M., Iturriaga L.B. (2017). Extraction and Physicochemical Characterization of Pectin from Tomato Processing Waste. J. Food Meas. Charact..

[82] Hua X., Wang K., Yang R., Kang J., Zhang J. (2015). Rheological Properties of Natural Low-Methoxyl Pectin Extracted from Sunflower Head. Food Hydrocoll..

[83] Busato B., de Almeida Abreu E.C., de Oliveira Petkowicz C.L., Martinez G.R., Rodrigues Noleto G. (2020). Pectin from Brassica Oleracea Var. Italica Triggers Immunomodulating Effects in Vivo. Int. J. Biol. Macromol..

[84] Maxwell E.G., Colquhoun I.J., Chau H.K., Hotchkiss A.T., Waldron K.W., Morris V.J., Belshaw N.J. (2016). Modified Sugar Beet Pectin Induces Apoptosis of Colon Cancer Cells via an Interaction with the Neutral Sugar Side-Chains. Carbohydr. Polym..

[85] Sucheta, Misra N.N., Yadav S.K. (2020). Extraction of Pectin from Black Carrot Pomace Using Intermittent Microwave, Ultrasound and Conventional Heating: Kinetics, Characterization and Process Economics. Food Hydrocoll..

[86] Kazemi M., Khodaiyan F., Hosseini S.S. (2019). Eggplant Peel as a High Potential Source of High Methylated Pectin: Ultrasonic Extraction Optimization and Characterization. LWT.

[87] Torkova A.A., Lisitskaya K.V., Filimonov I.S., Glazunova O.A., Kachalova G.S., Golubev V.N., Fedorova T.V. (2018). Physicochemical and Functional Properties of *Cucurbita maxima* Pumpkin Pectin and Commercial Citrus and Apple Pectins: A Comparative Evaluation. PLoS ONE.

[88] Ezzati S., Ayaseh A., Ghanbarzadeh B., Heshmati M.K. (2020). Pectin from Sunflower By-Product: Optimization of Ultrasound-Assisted Extraction, Characterization, and Functional Analysis. Int. J. Biol. Macromol..

[89] Kazemi M., Khodaiyan F., Hosseini S.S. (2019). Utilization of Food Processing Wastes of Eggplant as a High Potential Pectin Source and Characterization of Extracted Pectin. Food Chem..

[90] Song Y.R., Han A.R., Park S.G., Cho C.W., Rhee Y.K., Hong H. (2020). Do Effect of Enzyme-Assisted Extraction on the Physicochemical Properties and Bioactive Potential of Lotus Leaf Polysaccharides. Int. J. Biol. Macromol..

[91] Higuera-Coelho R.A., Lizarraga L., Ponce N.M.A., Stortz C.A., Rojas A.M., Bernhardt D.C., Fissore E.N. (2021). Novel Gelling Pectins from *Zea mays* Husks’ Agro-Industrial Residue and Their Interaction with Calcium and Iron (II). Bioact. Carbohydr. Diet. Fibre.

[92] Sabater C., Molina-Tijeras J.A., Vezza T., Corzo N., Montilla A., Utrilla P. (2019). Intestinal Anti-Inflammatory Effects of Artichoke Pectin and Modified Pectin Fractions in the Dextran Sulfate Sodium Model of Mice Colitis. Artificial Neural Network Modelling of Inflammatory Markers. Food Funct..

[93] Sabater C., Corzo N., Olano A., Montilla A. (2018). Enzymatic Extraction of Pectin from Artichoke (*Cynara scolymus* L.) by-Products Using Celluclast®1.5L. Carbohydr. Polym..

[94] Sabater C., Abad-García C., Delgado-Fernández P., Corzo N., Montilla A. (2020). Carbohydrate Fraction Characterisation of Functional Yogurts Containing Pectin and Pectic Oligosaccharides through Convolutional Networks. J. Food Compos. Anal..

[95] Idrovo Encalada A.M., De’Nobili M.D., Ponce A.N.M., Stortz C.A., Fissore E.N., Rojas A.M. (2021). Antioxidant Edible Film Based on a Carrot Pectin-Enriched Fraction as an Active Packaging of a Vegan Cashew Ripened Cheese. Int. J. Food Sci. Technol..

[96] Holland C., Ryden P., Edwards C.H., Grundy M.M.L. (2020). Plant Cell Walls: Impact on Nutrient Bioaccessibility and Digestibility. Foods.

[97] Dias do Nascimento Santos D.K., da Silva Barros B.R., da Cruz Filho I.J., da Silva Bezerra N., da Silva P.R., do Bomfim Nascimento P.H., do Carmo Alves de Lima M., Napoleão T.H., de Melo C.M.L. (2021). Pectin-like Polysaccharide Extracted from the Leaves of Conocarpus Erectus Linnaeus Promotes Antioxidant, Immunomodulatory and Prebiotic Effects. Bioact. Carbohydr. Diet. Fibre.

[98] Vriesmann L.C., de Oliveira Petkowicz C.L. (2017). Cacao Pod Husks as a Source of Low-Methoxyl, Highly Acetylated Pectins Able to Gel in Acidic Media. Int. J. Biol. Macromol..

[99] Mzoughi Z., Abdelhamid A., Rihouey C., Le Cerf D., Bouraoui A., Majdoub H. (2018). Optimized Extraction of Pectin-like Polysaccharide from *Suaeda fruticosa* Leaves: Characterization, Antioxidant, Anti-Inflammatory and Analgesic Activities. Carbohydr. Polym..

[100] Priyangini F., Walde S.G., Chidambaram R. (2018). Extraction Optimization of Pectin from Cocoa Pod Husks (*Theobroma cacao* L.) with Ascorbic Acid Using Response Surface Methodology. Carbohydr. Polym..

[101] Amorim J.C., Vriesmann L.C., Petkowicz C.L.O., Martinez G.R., Noleto G.R. (2016). Modified Pectin from Theobroma Cacao Induces Potent Pro-Inflammatory Activity in Murine Peritoneal Macrophage. Int. J. Biol. Macromol..

[102] Asgari K., Labbafi M., Khodaiyan F., Kazemi M., Hosseini S.S. (2020). Valorization of Walnut Processing Waste as a Novel Resource: Production and Characterization of Pectin. J. Food Process Preserv..

[103] Asgari K., Labbafi M., Khodaiyan F., Kazemi M., Hosseini S.S. (2020). High-Methylated Pectin from Walnut Processing Wastes as a Potential Resource: Ultrasound Assisted Extraction and Physicochemical, Structural and Functional Analysis. Int. J. Biol. Macromol..

[104] Kazemi M., Khodaiyan F., Labbafi M., Hosseini S.S. (2020). Ultrasonic and Heating Extraction of Pistachio By-Product Pectin: Physicochemical, Structural Characterization and Functional Measurement. J. Food Meas. Charact..

[105] Kazemi M., Khodaiyan F., Labbafi M., Saeid Hosseini S., Hojjati M. (2019). Pistachio Green Hull Pectin: Optimization of Microwave-Assisted Extraction and Evaluation of Its Physicochemical, Structural and Functional Properties. Food Chem..

[106] Hennessey-Ramos L., Murillo-Arango W., Vasco-Correa J., Astudillo I.C.P. (2021). Enzymatic Extraction and Characterization of Pectin from Cocoa Pod Husks (*Theobroma cacao* L.) Using Celluclast® 1.5 L. Molecules.

[107] Ma M., Long X., Wang Y., Chen K., Zhao M., Zhu L., Chen Q. (2024). Synergized Enzyme-Ultrasound-Assisted Aqueous Two-Phase Extraction and Antioxidant Activity Validation of Polysaccharides from Tobacco Waste. Microchem. J..

[108] Posé S., Kirby A.R., Paniagua C., Waldron K.W., Morris V.J., Quesada M.A., Mercado J.A. (2015). The Nanostructural Characterization of Strawberry Pectins in Pectate Lyase or Polygalacturonase Silenced Fruits Elucidates Their Role in Softening. Carbohydr. Polym..

[109] Dickinson E. (2003). Hydrocolloids at Interfaces and the Influence on the Properties of Dispersed Systems. Food Hydrocoll..

[110] Kazemi M., Amiri Samani S., Ezzati S., Khodaiyan F., Hosseini S.S., Jafari M. (2021). High-Quality Pectin from Cantaloupe Waste: Eco-Friendly Extraction Process, Optimization, Characterization and Bioactivity Measurements. J. Sci. Food Agric..

[111] Min B., Lim J., Ko S., Lee K.G., Lee S.H., Lee S. (2011). Environmentally Friendly Preparation of Pectins from Agricultural Byproducts and Their Structural/Rheological Characterization. Bioresour. Technol..

[112] Hwang J.K., Kim C.J., Kim C.T. (1998). Extrusion of Apple Pomace Facilitates Pectin Extraction. J. Food Sci..

[113] Maneerat N., Tangsuphoom N., Anadi N. (2017). Effect of Extraction Condition on Properties of Pectin from Banana Peels and Its Function as Fat Replacer in Salad Cream. J Food Sci Technol.

[114] Jiang Y., Du Y., Zhu X., Xiong H., Woo M.W., Hu J. (2012). Physicochemical and Comparative Properties of Pectins Extracted from *Akebia trifoliata* Var. *Australis* Peel. Carbohydr. Polym..

[115] Zhang L., Ye X., Ding T., Sun X., Xu Y., Liu D. (2013). Ultrasound Effects on the Degradation Kinetics, Structure and Rheological Properties of Apple Pectin. Ultrason. Sonochem.

[116] Salimi A., Khodaiyan F., Askari G., Hosseini S.S. (2024). A Zero-Waste Approach towards a Sustainable Waste Management of Apple: Extraction of Value-Added Products and Their Application as Edible Coating. Food Hydrocoll..

[117] Tsirigotis-Maniecka M., Szyk-Warszyńska L., Lamch Ł., Weżgowiec J., Warszyński P., Wilk K.A. (2021). Benefits of PH-Responsive Polyelectrolyte Coatings for Carboxymethyl Cellulose-Based Microparticles in the Controlled Release of Esculin. Mater. Sci. Eng. C.

[118] Tsirigotis-Maniecka M., Szyk-Warszyńska L., Maniecki Ł., Szczęsna W., Warszyński P., Wilk K.A. (2021). Tailoring the Composition of Hydrogel Particles for the Controlled Delivery of Phytopharmaceuticals. Eur. Polym. J..

[119] Hussain M., Bakalis S., Gouseti O., Zahoor T., Anjum F.M., Shahid M. (2015). Dynamic and Shear Stress Rheological Properties of Guar Galactomannans and Its Hydrolyzed Derivatives. Int. J. Biol. Macromol..

[120] Salehi F., Inanloodoghouz M., Karami M. (2023). Rheological Properties of Carboxymethyl Cellulose (CMC) Solution: Impact of High Intensity Ultrasound. Ultrason. Sonochem.

[121] Ro J., Kim Y., Kim H., Jang S.B., Lee H.J., Chakma S., Jeong J.H., Lee J. (2013). Anti-Oxidative Activity of Pectin and Its Stabilizing Effect on Retinyl Palmitate. Korean J. Physiol. Pharmacol..

[122] Rodrigues J.A.G., Vanderlei E.D.S.O., Silva L.M.C.M., De Araújo I.W.F., De Queiroz I.N.L., De Paula G.A., Abreu T.M., Ribeiro N.A., Bezerra M.M., Chaves H.V. (2012). Antinociceptive and Anti-Inflammatory Activities of a Sulfated Polysaccharide Isolated from the Green Seaweed Caulerpa Cupressoides. Pharmacol. Rep..

[123] Ye C., Han N., Teng F., Wang X., Xue R., Yin J. (2013). Extraction Optimization of Polysaccharides of Schisandrae Fructus and Evaluation of Their Analgesic Activity. Int. J. Biol. Macromol..

[124] Schepetkin I.A., Quinn M.T. (2006). Botanical Polysaccharides: Macrophage Immunomodulation and Therapeutic Potential. Int. Immunopharmacol..

[125] Wang H., Sakuragi Y., Pasternak T.P., Showalter A.M., Basu D. (2016). Extensin and Arabinogalactan-Protein Biosynthesis: Glycosyltransferases, Research Challenges, and Biosensors. Front. Plant Sci..

[126] de Oliveira A.F., do Nascimento G.E., Iacomini M., Cordeiro L.M.C., Cipriani T.R. (2017). Chemical Structure and Anti-Inflammatory Effect of Polysaccharides Obtained from Infusion of Sedum Dendroideum Leaves. Int. J. Biol. Macromol..

[127] Heinzel S., Marchingo J.M., Horton M.B., Hodgkin P.D. (2018). The Regulation of Lymphocyte Activation and Proliferation. Curr. Opin. Immunol..

[128] Zhang W., Xu P., Zhang H. (2015). Pectin in Cancer Therapy: A Review. Trends Food Sci. Technol..

[129] Du Toit A. (2014). FtsZ and FtsA Find the Right Place. Nat. Rev. Microbiol..

[130] Sikkema J., De Bont J.A.M., Poolman B. (1995). Mechanisms of Membrane Toxicity of Hydrocarbons. Microbiol. Rev..

[131] Ho Y.Y., Lin C.M., Wu M.C. (2017). Evaluation of the Prebiotic Effects of Citrus Pectin Hydrolysate. J. Food Drug Anal..

[132] WHO (2021). Human Health Risk Assessment Toolkit.

[133] Brownlee I.A. (2011). The Physiological Roles of Dietary Fibre. Food Hydrocoll..

[134] Lv Q.Q., Cao J.J., Liu R., Chen H.Q. (2021). Structural Characterization, α-Amylase and α-Glucosidase Inhibitory Activities of Polysaccharides from Wheat Bran. Food Chem..

[135] Cudrey C., van Tilbeurgh H., Gargouri Y., Verger R. (1993). Inactivation of Pancreatic Lipases by Amphiphilic Reagents 5-(Dodecyldithio)-2-Nitrobenzoic Acid and Tetrahydrolipstatin. Dependence upon Partitioning between Micellar and Oil Phases. Biochemistry.

[136] Stephens R.W., Arhire L., Covasa M. (2018). Gut Microbiota: From Microorganisms to Metabolic Organ Influencing Obesity. Obesity.

[137] Bianchi F., Larsen N., de Mello Tieghi T., Adorno M.A.T., Kot W., Saad S.M.I., Jespersen L., Sivieri K. (2018). Modulation of Gut Microbiota from Obese Individuals by in vitro Fermentation of Citrus Pectin in Combination with Bifidobacterium Longum BB-46. Appl. Microbiol. Biotechnol..

[138] Mortensen P.B., Holtug K., Rasmussen H.S. (1988). Short-Chain Fatty Acid Production from Mono- and Disaccharides in a Fecal Incubation System: Implications for Colonic Fermentation of Dietary Fiber in Humans. J. Nutr..

[139] Dalby M.J., Ross A.W., Walker A.W., Morgan P.J. (2017). Dietary Uncoupling of Gut Microbiota and Energy Harvesting from Obesity and Glucose Tolerance in Mice. Cell Rep..

[140] Heidary Moghaddam R., Samimi Z., Moradi S.Z., Little P.J., Xu S., Farzaei M.H. (2020). Naringenin and Naringin in Cardiovascular Disease Prevention: A Preclinical Review. Eur. J. Pharmacol..

